# White Mn-MOF nanozymes with peroxidase-activity specificity overcome color and O_2_ effects on colorimetric test strips

**DOI:** 10.1038/s42004-025-01772-z

**Published:** 2025-12-04

**Authors:** Lei Han, Jingying Tan, Yucui Zhang

**Affiliations:** College of Chemistry and Pharmaceutical Sciences, Qingdao Agricultural University, Qingdao, Shandong China

**Keywords:** Bioanalytical chemistry, Two-dimensional materials, Metal-organic frameworks, Heterogeneous catalysis

## Abstract

Given the promising prospect of nanozymes in colorimetric test strips, it is essential to eliminate the interferences of their multi-activities and various colors on the test strip. Here, white Mn-based metal-organic frameworks (Mn-MOFs) with ultrathin 2D morphology (3 nm thick) were successfully synthesized by a simple ultrasonic approach. The origin of the white optical property in Mn-MOFs was systematically investigated, revealing that it stems from specific metal-ligand coordination polymerization rather than morphological features or defect states. Mn-MOF nanozymes possessed exclusive peroxidase-mimicking activity rather than oxidase-like activity, effectively resisting O_2_ interference during colorimetric assay. Moreover, Mn-MOF nanozymes displayed unique substrate selectivity without additional modification. Unlike other colored nanozymes, the whiteness of Mn-MOF nanozymes enhanced the paper’s whiteness, boosting contrast for colorimetric detection on test strip. This study pioneers a systematic investigation into the origin of whiteness in MOF nanozymes. The coordination-defined properties enable interference-free optical design and O_2_-resistant on-site detection.

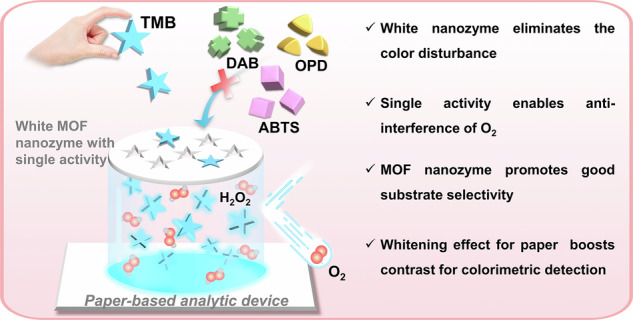

## Abstract (web summary)

Nanozymes have shown promise in the development of portable colorimetric test strips, but their inherent colours typically interfere with the colorimetric readouts, while oxidase-like activity often disturbs measurements made under an aerobic atmosphere. Here, the authors develop an optically white Mn-MOF nanozyme with exclusively peroxidase-mimicking activity, demonstrating high sensitivity and inherent substrate selectivity in real-world detection applications.

## Introduction

So far, enzymes have been widely applied as common recognition elements in analytical platforms, due to their high activity and selectivity^1,2^. Nevertheless, enzymes generally face some application bottlenecks, such as complex purification process, poor stability and high cost^3–5^. Recently, nanozymes (nanomaterials with enzyme-like activities)^6–9^, have emerged as a promising alternative to traditional enzymes in diagnostics, detection and sensing, owing to their high catalytic activity, easy preparation, good stability, and low cost^10–16^. However, compared to natural enzymes, the relatively weak reaction selectivity of nanozymes still poses significant challenges to the analytical applications^17–19^. For example, peroxidase-like nanozymes, one of the most common subfamily of nanozymes, are widely used in colorimetric analysis, where they catalyze the oxidation of chromogenic substrate with H_2_O_2_ to produce a chromogenic product^18,20,21^. Nevertheless, many peroxidase-like nanozymes also possess oxidase-like activity, such as V_2_O_5_^22^, Co_3_O_4_^23^, MnO_2_^24^, CeO_2_^25^, CuO^26^, Co_3_V_2_O_8_^27^, Pt^28^, Pd@Ir^29^, Fe/Co-based metal-organic framework (Fe/Co-MOF)^30^, Au-MOF^31^, Pt-MOF^32^, and Prussian Blue^33^. This will cause the chromogenic substrates to be directly oxidized by O_2_ in the air in the absence of H_2_O_2_. Moreover, the concentration of O_2_ in various real samples differs and fluctuates dynamically, which disrupts the colorimetric assay results based on peroxidase-like activity^34^. Therefore, it is crucial to explore the peroxidase-like nanozymes without oxidase-like activity for the colorimetric analytic methods.

At present, known nanozymes generally lack inherent substrate specificity^35,36^. During the analysis of real samples, the certain coexisting organic contaminants (such as methylene blue^37^, rhodamine B^38^ and p-nitriphenol^39^) may act as competing substrates against the target substrate, potentially interfering with the detection accuracy. To achieve optimal substrate selectivity, researchers have explored several modification techniques, such as molecular imprinting^35,40^, and surface modification^41^. For example, Liu’s group utilized molecular imprinting technology to achieve high selectivity to chromogenic substrate^42^. However, these methods often involve the complicated operations, the reduced activity and even the structural damage of nanozymes, especially MOF-based nanozymes^40^. Considering that some MOFs can selectively adsorb certain molecules, MOF nanozymes are anticipated to achieve substrate selectivity without the need for additional modification. Nonetheless, known MOFs are rarely involved in the selectivity of catalytic activity, especially enzyme-like activity^43,44^. Thus, it is of great significance to discover MOF nanozymes with inherent substrate selectivity to address the poor selectivity of nanozymes.

Nanozymes have found widespread applications in the diverse colorimetric platforms, including solution assays, test strips, and microfluidic chips^45–47^. In particular, the powerful combination of nanozymes with colorimetric test strips has significantly reduced detection costs while improving storage, stability and portability, making their widespread adoption more feasible^20,48^. On nanozyme-based colorimetric test strips, nanozymes catalyze the chromogenic reaction to produce a visible color result^18,20^. Unfortunately, known nanozymes generally have various colors, such as yellow (V_2_O_5_^49^, CeO_2_^50^ and Ce-based metal-organic frameworks (Ce-MOF)^51^, black (Co_3_O_4_^52^ and Fe_3_O_4_^53,54^), brown (CuO^55^, MnO_2_^56^, Pd^31^), red (Au)^57^, pink (Co-MOF)^31^, blue (Prussian Blue)^29^ and orange (Fe-MOF)^58^. Although the abundant and uniform distribution of nanozymes on white paper can promote activity and amplify signal, the diversity of colors can interfere with the judgment of the chromogenic results on white paper. Therefore, it is fascinating to study how to avoid the color interference of nanozymes and even enhance the whiteness of paper. This inspired us to excavate white nanozymes and explore their applications on test strips.

**Fig. 1:**
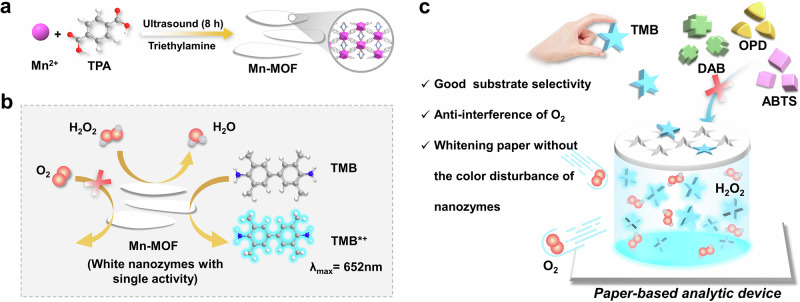
Design of white Mn-MOF nanozymes with single peroxidase-like activity for test strip. **a** Schematic preparation of Mn-MOF nanozymes. **b** Single peroxidase-like activity of Mn-MOF nanozymes unaffected by O_2_. **c** Colorimetric test strip based on Mn-MOF nanozymes.

Here, we facilely synthesized the white ultra-thin Mn-MOF nanosheets (NSs) using a simple ultrasonic method (Fig. 1a). Remarkably, Mn-MOF NSs exhibit excellent whiteness, the origin of which has been systematically investigated. Notably, Mn-MOF NSs not only exhibited enzyme-like activity but also demonstrated two levels of catalytic specificity: (1) Reaction selectivity: It possessed peroxidase-like activity but not oxidase-like activity, thereby preventing the interference of O_2_ during detection (Fig. 1b). (2) Substrate selectivity: It also exhibited inherent substrate specificity without further modification, as it could specifically oxidize substrate 3,3’,5,5’-tetramethylbenzidine (TMB) while other substrates remained unaffected (Fig. 1c). Furthermore, we found an ideal application for these white nanozymes—white nanozyme test strips. Excitingly, unlike other colored nanozymes, the whiteness of Mn-MOF nanozymes not only avoided color interference from the nanozymes, but also strengthened the whiteness of paper, highlighting color changes on the test strip and thus improving sensitivity. Further, the constructed H_2_O_2_ test strip showed high sensitivity, strong anti-interference capability in aerobic environment, and excellent store stability (Fig. 1c). To demonstrate its versatility, we further extended the use of nanozymes for detecting glucose and sarcosine. This study is anticipated to stimulate further exploration of the unique whiteness and catalytic properties of MOF nanozymes for advanced colorimetric detection applications.

## Results

### Synthesis and characterization of Mn-MOF nanozymes

**Fig. 2:**
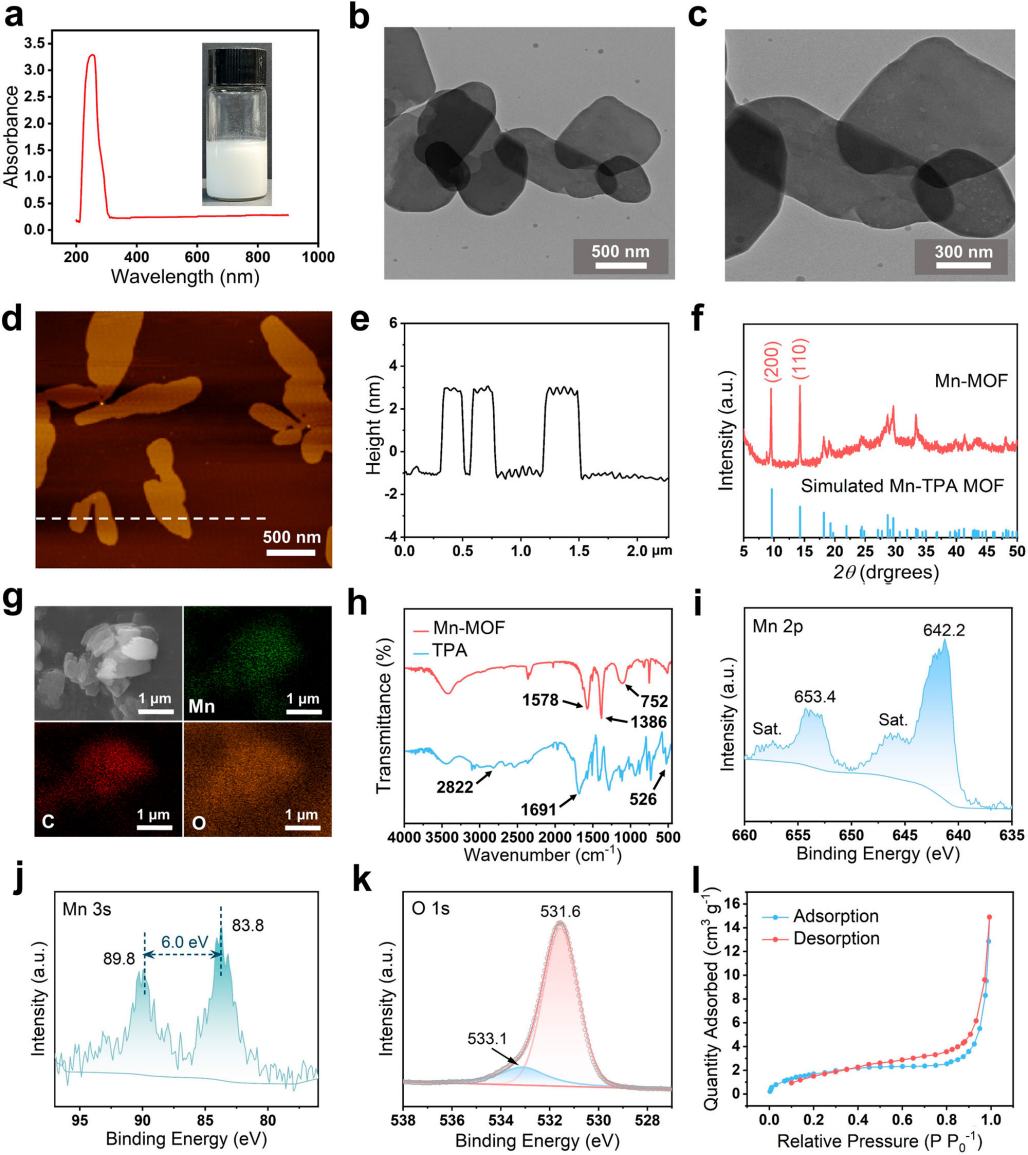
Characterization of Mn-MOF nanozymes. **a** UV–vis spectrum and photograph of Mn-MOF suspension (1 mg mL^−1^). **b**, **c** TEM images of Mn-MOF. AFM image (**d**) and (**e**) corresponding height profile (**e**) of Mn-MOF. **f** XRD pattern of Mn-MOF and simulated Mn-TPA MOF. **g** SEM image and EDX elemental mapping images of Mn-MOF. **h** FT-IR spectra of Mn-MOF and TPA. High-resolution XPS patterns for (**i**) Mn 2p, (**j**) Mn 3 *s* and (**k**) O 1s of Mn-MOF. **l** Nitrogen absorption/desorption isotherm of Mn-MOF.

In view of the fact that the most of MOF nanozymes were synthesized by hydrothermal approach, which requires high temperature, high pressure and sealed equipment, we tried to synthesize Mn-MOF by facile ultrasonic method (Fig. 1a). MnCl_2_ and terephthalic acid (TPA) were rapidly added into the solution containing dimethyl formamide (DMF) and triethylamine (TEA). After ultrasonic processing, a uniform white solution was produced, and white Mn-MOF precipitates were obtained by centrifugation and vacuum drying (Fig. 2a). Significantly, white Mn-MOF had no absorption peak within 300–900 nm (Fig. 2a), which will facilitate the chromogenic assay results.

The morphology of white products was observed by transmission electron microscopy (TEM). They exhibited two-dimension (2D) nanosheet structure with smooth edges (Fig. 2b, c). Statistical analysis revealed that the Mn-MOF NSs exhibit lengths ranging from 0.28 to 1.99 μm and widths between 0.18 and 1.46 μm (Supplementary Table 1). Overall, the size of Mn-MOF NSs was predominantly in the range of 0.2–1 μm (Supplementary Fig. 1). More detailed information about morphology and size of Mn-MOF was researched by atomic force microscope (AFM). Mn-MOF nanosheets displayed a smooth surface with an average thickness of 3 nm (Fig. 2d, e), confirming the ultra-thin 2D structure. X-ray diffractometer (XRD) pattern of Mn-MOF (Fig. 2f) was consistent with the previously reported monoclinic crystalline framework, Mn_4_(TPA)_4_(H_2_O)_8_^59^, and the well-resolved peaks indicated high crystallinity. To better demonstrate the advantages of the ultrasonic synthesis method, we adopted the traditional hydrothermal method at 150 °C to prepare Mn-MOF^59^, which resulted in micron-sized, solid, irregular chunks (Supplementary Fig. 2a, b). This comparison highlights the advantage of our synthesis approach, which not only eliminates the need for high-temperature heating and sealed equipment, but also successfully produces ultra-thin 2D nanostructured morphology.

Chemical composition (Mn, O, and C) and uniform distribution of Mn-MOF are affirmed by energy dispersive X-ray (EDX) spectroscopy (Fig. 2g). The Fourier transform infrared (FT-IR) spectra (Fig. 2h) reflected that the characteristic beaks of Mn-MOF were different with ligand TPA. The peaks of TPA at 2822, 1691 and 526 cm^−1^ conformed to *ν*(OH), *ν*(C=O) and *δ*(C=O) of the nonionized carboxyl groups. As for Mn-MOF, the above peaks disappeared, while new peaks were generated at 1578 and 1386 cm^−1^, which were correspond to asymmetric and symmetrirc stretching vibrations of –COO^−^_._ These results demonstrated the deprotonation of acidic carboxyl, attributing to the complexation of manganese ions and carboxyl groups. Moreover, the appearance of characteristic peak at 752 cm^−1^ indicated that the 1,4-substituent bond core of TPA turned into the ring-out-of-plane vibration, indicating the effective coordination between the manganese ions and TPA. To further determine the surface elements and the oxidation state of manganese, X-ray photoelectron spectroscopy (XPS) was conducted. The XPS survey spectra of Mn-MOF (Supplementary Fig. 3a) suggest the existences of Mn, C, and O elements. The Mn 2p XPS spectrum of Mn-MOF also displayed evident peaks at 642.2 and 653.4 eV (Fig. 2i), coinciding with Mn2p_3/2_ of Mn^2+^ in Mn‒O bonds, different from the peaks of MnO^60^. Fitting the Mn 2p spectrum alone can be challenging and sometimes unreliable for unequivocally determining the oxidation state. To address this conclusively, we relied on the Mn 3 *s* multiplet splitting energy Mn oxidation states can be distinguished by the spin–orbit peak splitting of Mn 3 *s*, a well-established technique for identifying the oxidation states of manganese^61^. The principle is that the splitting energy value systematically decreases with increasing oxidation state: it is approximately 6.0 eV for Mn^2+^ and typically ranges from 4.7 to 4.9 eV for Mn^4+^^59^. As shown in Fig. 2j, the peak splitting value of Mn 3 *s* was measured to be 6.0 eV, which is consistent with Mn^2+^ and distinctly different from the value expected for Mn^4+^. This provides strong evidence that manganese in Mn-MOF nanozymes is in the +2 oxidation state without detectable Mn^4+^. Meanwhile, the fitting characteristic peaks of O 1 *s* at 531.6 and 533.1 eV (Fig. 2k) corresponded to the Mn-carbonate/C=O and C-O bonds. Particularly, the peak of O 1 *s* at 531.6 eV was not characteristic of metal oxides. Moreover, the C 1 *s* spectrum of Mn-MOF could be deconvoluted into three peaks at 284.8, 286.3 and 288.5 eV (Supplementary Fig. 3b), which were typical values of C-C/C=C, C-O and O=C-O, respectively^56^, indicating the existence of TPA. The quantitative elemental ratios based on XPS analysis indicated that the composition of Mn-MOF nanozymes consisted of 5.36% Mn 2p, 26.72% O 1 *s*, and 62.92% C 1 *s* (Supplementary Table 2). These above results indicated white 2D ultra-thin Mn-MOF nanosheets were successfully synthesized by facile ultrasonic approach.

The mesoporous properties of Mn-MOF nanozymes were studied by nitrogen adsorption/desorption isotherm (BET). The isotherm shows IV-type and H_3_-type hysteresis loop (*P* *P*_0_^−1^ > 0.4) (Fig. 2l), indicating that Mn-MOF had mesoporous properties. The BET specific surface area of Mn-MOF was 6.5109 m^2^ g^−1^. In addition, the pore size distribution was analyzed by Barrett–Joyner–Halenda method in the desorption part and the average pore size was 14.8 nm. The high specific surface area will be conducive to catalysis of Mn-MOF.

### Origin of whiteness in Mn-MOF

**Fig. 3:**
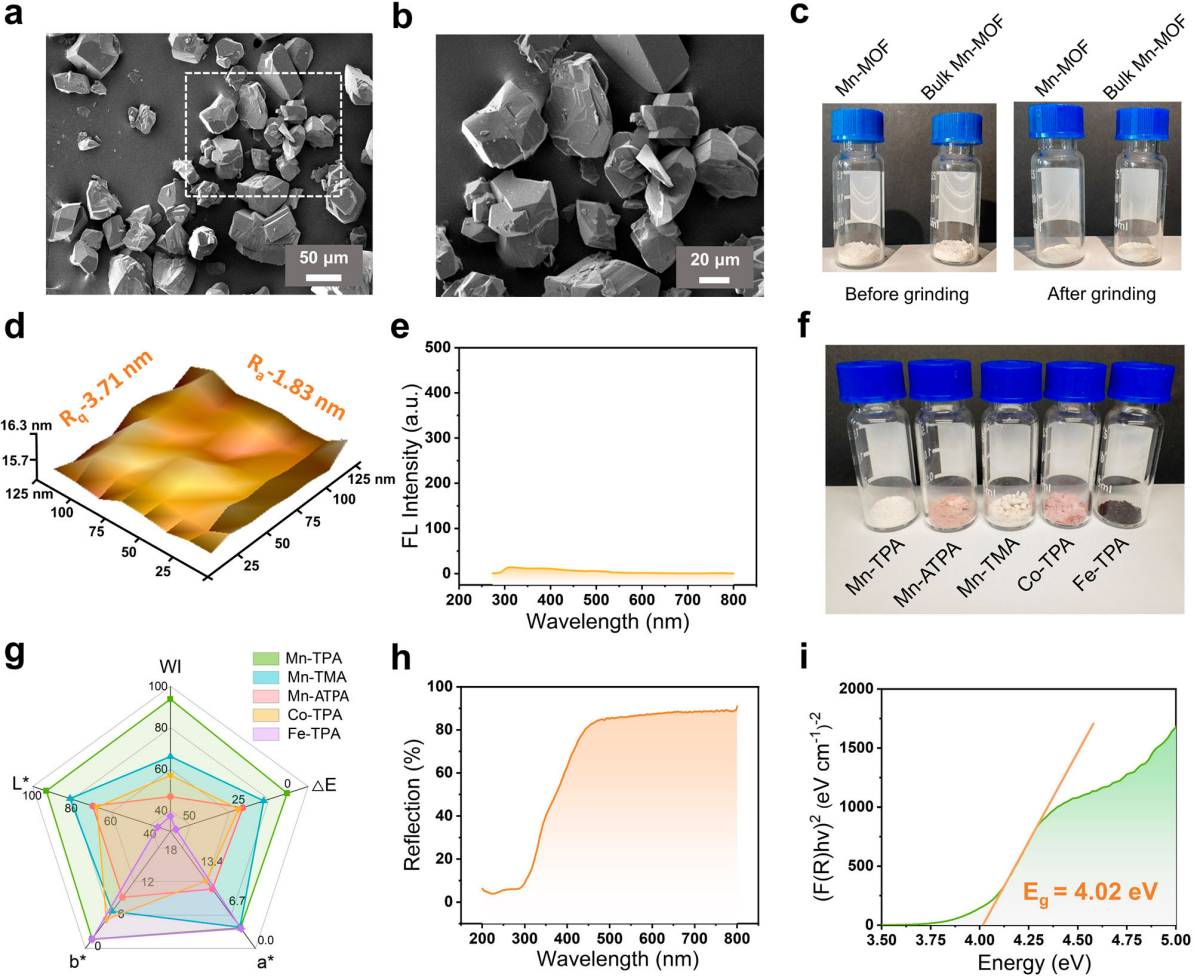
Origin study of the whiteness of Mn-MOF. **a** SEM images of hydrothermally synthesized bulk Mn-MOF. **b** Enlarged view of the pane-marked areas of (**a**). **c** Photographs of Mn-MOF NSs and bulk Mn-MOF before and after mechanical grinding. **d** AFM topographic image of MOF NSs for surface roughness assay. **e** Fluorescence spectrum of Mn-MOF NSs under 253 nm excitation. **f** Photographs of various MOF prepared from different metal ions (Mn^2+^, Fe^3+^ and Co^2+^) and ligands (TPA, ATPA and TMA) under identical conditions. **g** Radar chart comparing whiteness parameters (L*, a*, b*, *WI*, and Δ*E*) of various MOFs. **h** UV–Vis DRS of MOF NSs. **i** Tauc plot derived from DRS data for direct bandgap estimation.

The synthesized Mn-MOF exhibited white coloration, which holds significant importance for white test strip substrates. Therefore, this stimulates us to systematically investigated the origin of this whiteness and quantitatively evaluated its degree. Typically, whiteness arises from three primary mechanisms: (1) nanostructure-induced scattering effects, (2) defect-state luminescence, or (3) intrinsic metal/ligand characteristics^62–67^. The hydrothermally synthesized bulk Mn-MOF showed no apparent nanostructures (Fig. 3a, b), yet remained white (Fig. 3c). Moreover, both bulk Mn-MOF and ultrasonically synthesized Mn-MOF NSs maintained their white color after grinding (Fig. 3c), eliminating nanostructure scattering as the origin. Further, the surface roughness of Mn-MOF nanosheets was measured by AFM. The arithmetic average roughness (*R*_*a*_) and root mean square roughness (*R*_*q*_) were determined to be 1.83 nm and 3.71 nm, respectively, both significantly below 10 nm. This result excludes surface roughness-enhanced scattering as the origin of whiteness. (Fig. 3d)^65^. Furthermore, visual inspection confirmed the absence of metallic luster, ruling out specular reflection. Additionally, fluorescence spectra revealed no detectable emission peaks within 300–800 nm under 253 nm excitation (the maximum absorption wavelength), which rules out defect-state luminescence as a possible cause of the white coloration (Fig. 3e)^64,68^.

These above results strongly suggested metal/ligand characteristics as the whiteness origin. To investigate the metal ion dependence, we substituted Mn^2+^ with Fe^3+^ and Co^2+^ while keeping all other synthesis conditions unchanged. As shown in Fig. 3f, the resulting MOFs exhibited pink and black colors, respectively. Ligand substitution of TPA with 2-aminoterephthalic acid (ATPA) or trimesic acid (TMA) produced pink and beige Mn-MOFs (Fig. 3f). Whiteness thus depends critically on both metal ion and ligand selection, where white coloration achieved exclusively through Mn^2+^-TPA coordination.

Subsequently, quantitative colorimetric evaluation was performed on these materials (Fig. 3g). Among all MOF samples, only the Mn-TPA MOF demonstrated a luminance value (L*) exceeding 90 (93.09) and a whiteness index (*WI*) above 90 (93.78), showing a clear distinction in the colorimetric results. In addition, this also confirmed that neither solvent/crystallization water nor synthesis methods contributed to the whiteness. According to international standards, *WI* > 90 qualifies as high whiteness. Compared to standard white TiO_2_^62^. The total color difference (Δ*E*) was <1 (Fig. 3g and Supplementary Table 3), indistinguishable to the naked eye, confirming superior whiteness. UV-Vis diffuse reflectance spectroscopy (DRS) of Mn-TPA MOF nanozymes showed about 90% reflectance within 450–800 nm (Fig. 3h), indicating no visible light absorption^69^. Moreover, The calculated bandgap of 4.02 eV (Fig. 3i) exceeded the 3.1 eV threshold for visible light absorption^62^. Taken together, the whiteness of Mn-MOF originates from specific metal–ligand combinations rather than nanostructures or defects, exhibiting no visible light absorption and producing high diffuse reflectance that generates its white appearance.

### Peroxidase-like activity and reaction specificity of Mn-MOF nanozymes

**Fig. 4:**
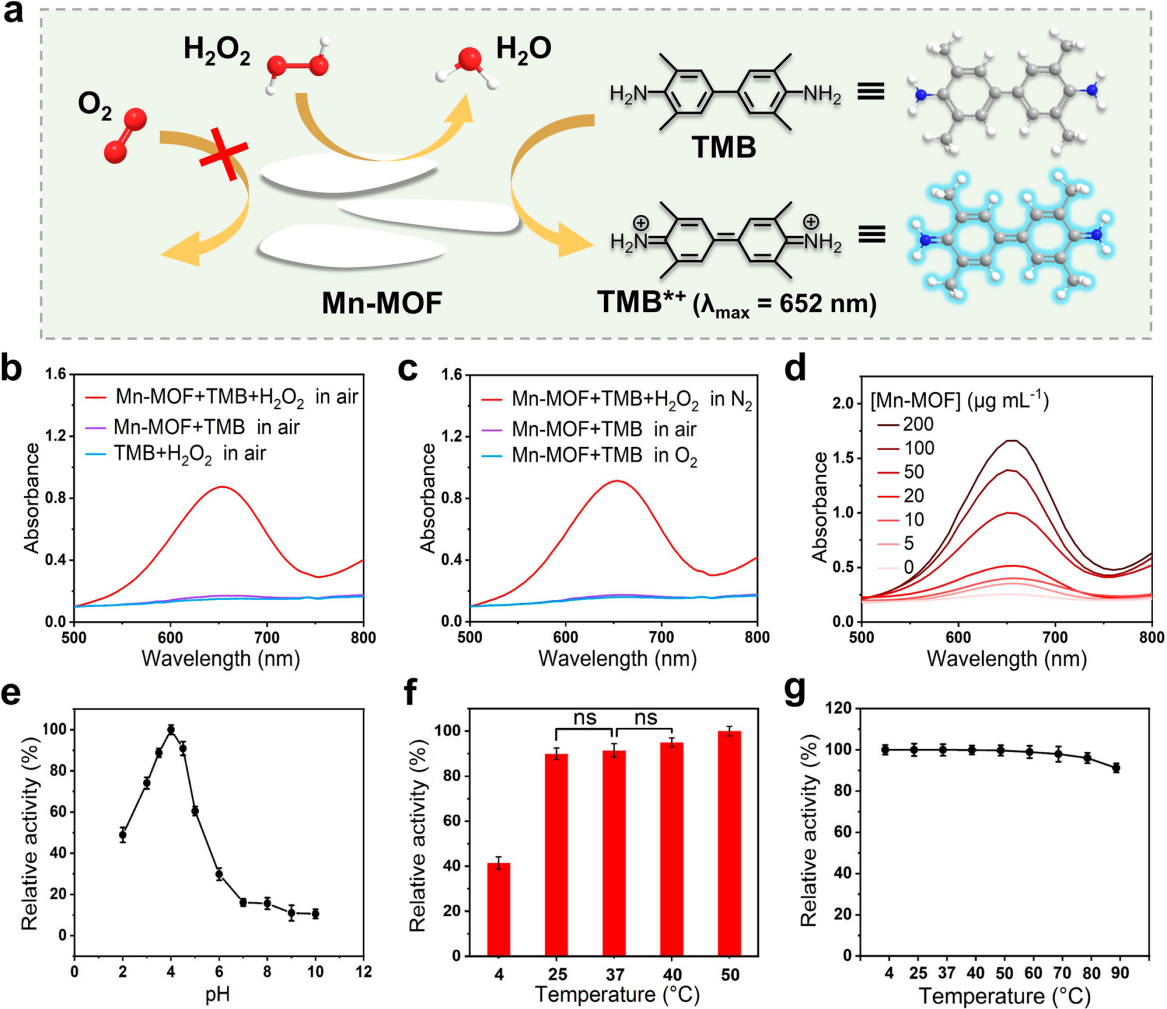
Peroxidase-like activity of Mn-MOF nanozymes. **a** Schematic of the catalytic activity of Mn-MOF nanozymes. **b** Typical UV–vis absorption spectra of catalytic oxidation of TMB by Mn-MOF nanozymes (50 μg mL^−1^) in the acetate buffer (pH 4.0, 0.1 m) in air. **c** Typical absorption spectra of TMB oxidation catalyzed by Mn-MOF nanozymes (50 μg mL^−1^) in different atmospheres. **d** Absorption spectra of TMB oxidation by H_2_O_2_ under the catalysis of different concentrations of Mn-MOF nanozymes. The influences of (**e**) pH and (**f**) temperature on the peroxidase-like activity of Mn-MOF nanozymes. ns, no significance. **g** The thermal stability of Mn-MOF nanozymes. Error bars: mean ± standard deviation (SD, *n* = 3).

To verify the peroxidase-like activity of Mn-MOF, chromogenic substrate TMB and H_2_O_2_ were added into Mn-MOF-catalyzed reaction solution in air. The characteristic absorbance peaks of TMB oxide (TMB*^+^) at 652 nm could be observed in TMB-H_2_O_2_-MOF system, while no obvious absorbance peaks appeared in the absence of Mn-MOF or H_2_O_2_ (Fig. 4a, b). The above results suggest Mn-MOF possesses inherent peroxidase-like activity but no oxidase-like activity. In addition, neither Mn^2+^ nor TPA had peroxidase-mimicking activity (Supplementary Fig. 4), indicating the peroxidase-like activity produced after the formation of the framework structure.

To further prove that Mn-MOF nanozymes have no oxidase-like activity, nanozymes-catalyzed reaction system was conducted in different concentrations of O_2_. Apparently, TMB could not be oxidized in the air- and even O_2_-saturated solutions without H_2_O_2_ (Fig. 4c). In contrast, there was a high absorption peak of TMB*^+^ in N_2_-saturated solution containing H_2_O_2_ (Fig. 4c), which was as high as that in air-saturated solution containing H_2_O_2_ (Fig. 4b). These results demonstrated the excellent reaction specificity of Mn-MOF nanozymes, i.e., single peroxidase-like activity without oxidase-like activity. Therefore, H_2_O_2_ is necessary for Mn-MOF-catalyzed reaction, and O_2_ cannot disturb it.

Like natural peroxidase, the peroxidase-mimicking activity of Mn-MOF exhibited Mn-MOF concentration-, pH- and temperature dependences (Fig. 4d–f). The absorption peak at 652 nm increased with the concentration of Mn-MOF nanozymes (Fig. 4d and Supplementary Fig. 5), confirming its peroxidase-like activity. Mn-MOF nanozymes exhibited strong activity under weakly acidic condition, and reached highest activity at pH 4.0 (Fig. 4e). The activity of Mn-MOF showed noticeable rise from 4 °C to 25 °C, and remained stable in a wide temperature range (25–40 °C) (Fig. 4f). Considering the activity of Mn-MOF at 25 °C was similar with those at higher temperatures (Fig. 4f), the subsequent experiments were conducted at 25 °C. In addition, Mn-MOF possessed good thermal stability, and remained over 90% of original activity at even 90 °C (Fig. 4g). Moreover, Mn-MOF nanozymes still exhibited almost unchanged peroxidase-like activity and good dispersibility after the storage for 6 months at room temperature (Supplementary Fig. 6), showing high storage stability.

### Steady-state kinetics mechanism of Mn-MOF nanozymes

The steady-state kinetics of Mn-MOF nanozymes were studied by varying the concentrations of TMB and H_2_O_2_, respectively (Supplementary Fig. 7). The experimental data conformed to the Michaelis-Menten equation. Moreover, as an important parameter for evaluating enzyme kinetics, the value of *K*_m_ is inversely correlated with the affinity of substrates with enzyme. The *K*_m_ value of Mn-MOF nanozymes for TMB (0.31 mm) was similar and lower than those of natural enzymes and some other nanozymes, and the *K*_m_ value of Mn-MOF nanozymes for H_2_O_2_ (0.063 mm) were 1–4 order of magnitudes lower than those of natural enzymes and some other nanozymes (Supplementary Table 4)^6,19,70–75^, which indicated the good affinity of Mn-MOF nanozymes with both substrates.

**Fig. 5:**
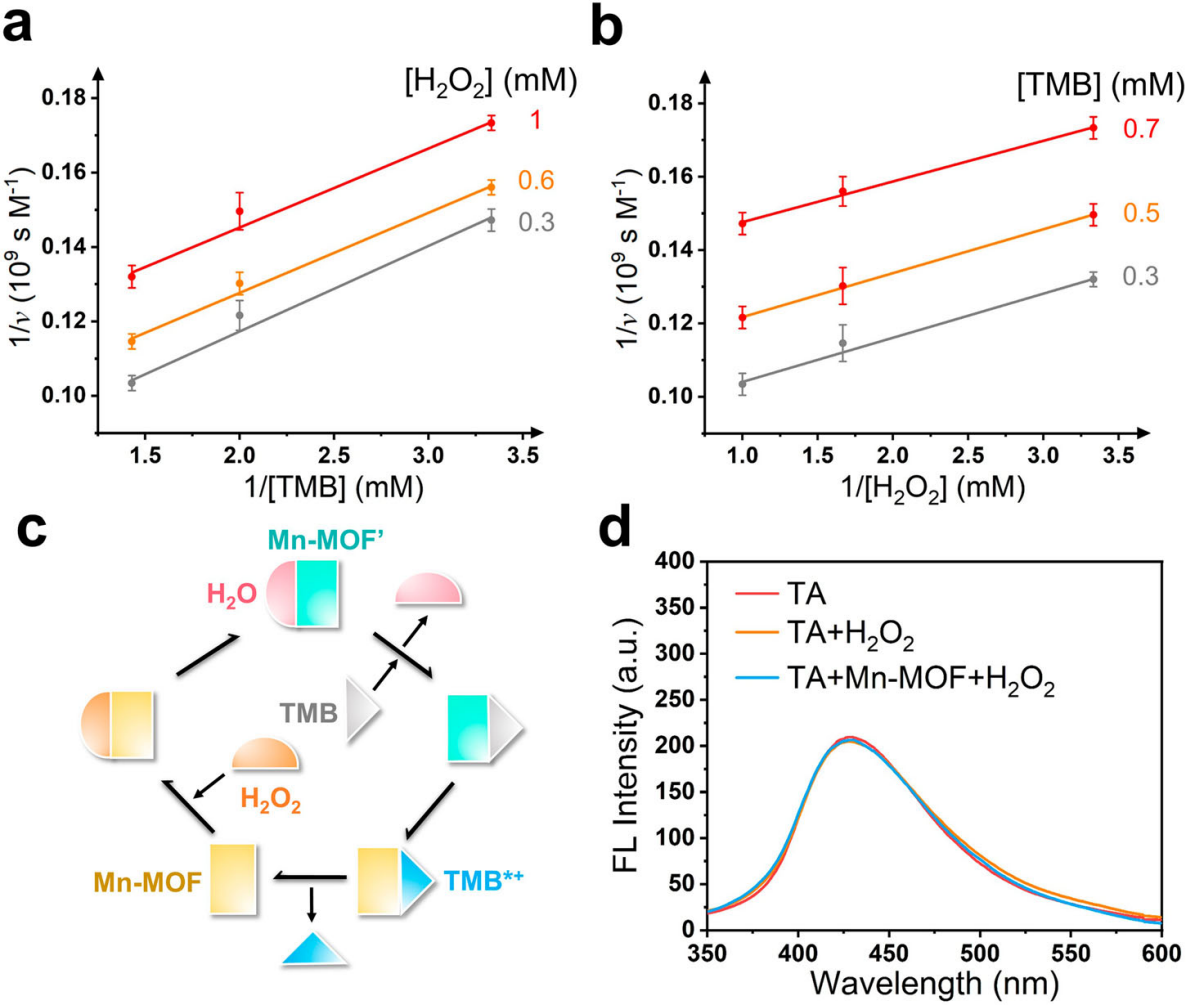
Steady-state kinetics of Mn-MOF nanozymes. The Lineweaver-Burk plots for various concentrations of (**a**) H_2_O_2_ (0.3, 0.6 and 1 mm) and (**b**) TMB (0.3, 0.5 and 0.7 mm). Error bars: mean ± SD (*n* = 3). **c** Schematic diagram of Ping-Pong catalytic mechanism. **d** Fluorescence (FL) spectra of different reaction solutions containing TA.

In order to investigate the catalytic kinetics mechanism of Mn-MOF nanozymes, the Lineweaver-Burk plots were drawn (Fig. 5a, b). The parallel double reciprocal plots demonstrated the Ping-Pong reaction mechanism. In other words, H_2_O_2_ and TMB reacted with Mn-MOF one by one. After the first product H_2_O was released, TMB reacted with Mn-MOF to produce TMB*^+^ (Fig. 5c). So, the catalysis may not be derived from the generation of hydroxyl radicals (·OH). To certify the above inference, terephthalic acid (TA) was chosen as a probe to track ·OH. As shown in Fig. 5d, the presence of Mn-MOF and H_2_O_2_ cannot affect the generation of the fluorescence product of TA, indicating that there is no formation of ·OH during the catalytic reaction. This was different with some enzymes and nanozymes, such as peroxidase^6^, Ni-MOF nanozymes^73^, Fe-MOF nanozymes^76,77^, Fe_3_O_4_ nanozymes^78^. This will provide the possibility for substrate selectivity of Mn-MOF nanozymes.

### Discovering intrinsic substrate specificity of Mn-MOF nanozymes

**Fig. 6:**
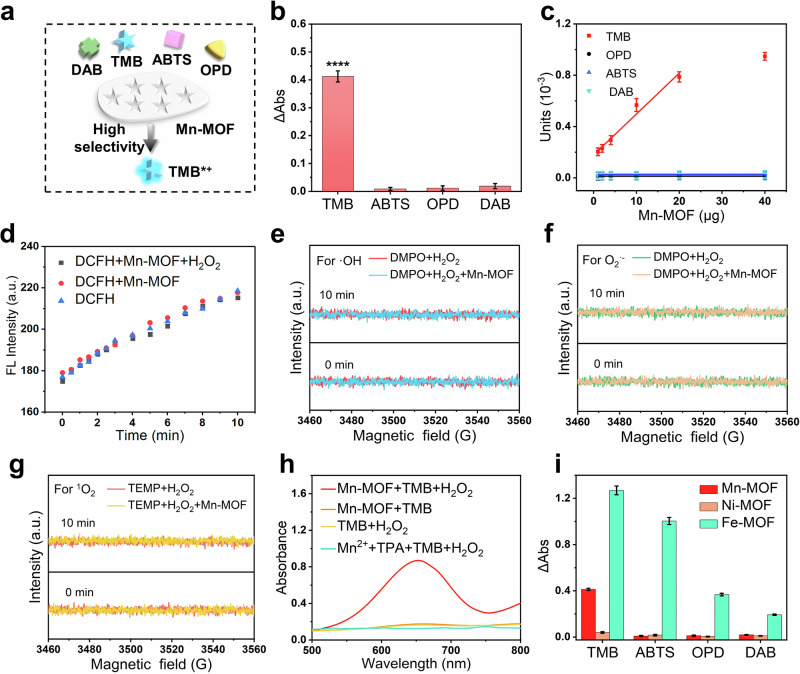
Substrate specificity of peroxidase-like activity. **a** Schematic illustration of the substrate selectivity of Mn-MOF nanozymes. **b** Peroxidase-like activity of Mn-MOF nanozymes to various substrate. **** *P* < 0.0001. **c** Activity units of different concentrations of Mn-MOF nanozymes for various substrates. **d** Fluorescence spectra of reaction solutions containing Mn-MOF nanozymes, H_2_O_2_, and fluorescent probe DCFH-DA. **e–g** ESR spectra for the detection of ROS generated by Mn-MOF nanozymes at catalytic reaction times of 0 min and 10 min: **e** ·OH trapped with DMPO; **f** O_2_·^−^ trapped with DMPO; **g** ^1^O_2_ trapped with TEMP. **h** Absorption spectra of catalytic oxidation of TMB by Mn-MOF nanozymes and controls in the presence of H_2_O_2_. **i** Peroxidase-like activity of various MOF nanozymes towards different substrates. Error bars: mean ± SD (*n* = 3).

As mentioned above, Mn-MOF nanozymes can catalyze the oxidation of TMB. So, other common substrates including 2, 2’-azino-bis (3-ethylbenzo-thiazoline-6-sulfonic acid) diammonium salt (ABTS), *o*-Phenylenediamine (OPD), and diaminobenzidine (DAB) were used to replace TMB (Fig. 6a). Surprisingly, Mn-MOF nanozymes could not catalyze the oxidation of ABTS, OPD and DAB in the presence of H_2_O_2_ (Fig. 6b and Supplementary Fig. 8), indicating that the peroxidase-like activity of Mn-MOF nanozymes possesses excellent substrate selectivity to TMB. In addition, Mn-MOF also could not catalyze the oxidation of the other three substrates (ABTS, OPD, and DAB) without H_2_O_2_ in air and O_2_ atmospheres (Supplementary Fig. 9), demonstrating Mn-MOF nanozymes really do not have oxidase-mimicking activity. To further confirm the substrate selectivity, the linear relationship between the activity unit (U) and the concentration of Mn-MOF nanozymes was investigated (Fig. 6c). The slopes of the fitted lines represented the specific activity of Mn-MOF nanozymes for different substrates, indicating good substrate selectivity of Mn-MOF to TMB. Besides, the specific peroxidase-like activity (0.03175 U mg^−1^) of Mn-MOF nanozymes was 1–2 orders of magnitude higher than those of previously reported Mn-based nanozymes (Supplementary Table 5), indicating the excellent catalytic activity of Mn-MOF nanozymes. Taken together, Mn-MOF nanozymes showed rare substrate selectivity, which is different and superior to other nanozymes and enzymes^18,19,35,78^.

Many POD-like nanozymes work by reactive oxygen species (ROS) as intermediates, and the oxidation of organic compounds by ROS usually lacks substrate selectivity^77,78^. In this work, we found that total ROS did not form during the catalytic process, by using 2’,7’-dichlorofluorescin diacetate (DCFH-DA) as the fluorescent probe of broad-spectrum ROS (Fig. 6d). To further investigate the potential generation of ROS by Mn-MOF nanozymes, we have conducted electron spin resonance (ESR) spectroscopy. 5,5-Dimethyl-1-pyrrolidine-*N*-oxide (DMPO) was used as the trapping agent for hydroxyl radicals (·OH) and superoxide radicals (O_2_·^−^), and 2,2,6,6-tetramethylpiperidine (TEMP) was used as the trapping agent for singlet oxygen (^1^O_2_). All the ESR results showed that, compared with the control group, no significant changes were observed for the Mn-MOF nanozymes. This indicated that no measurable ROS (·OH, O_2_·^⁻^ and ^1^O_2_) were generated during the catalytic process, which was consistent with the detection results of DCFH (Fig. 6d) and TA (Fig. 5d). This provides the possibility for the substrate selectivity.

To explore the reasons for the substrate specificity, the peroxidase-like activities of the precursors (MnCl_2_ and TPA) of Mn-MOF nanozymes were researched. The precursors cannot catalyze the oxidation of TMB, but peroxidase-mimicking activity arose after the formation of the frame structure (Fig. 6h and Supplementary Fig. 4). Subsequently, MOFs based on TPA and other metal ions (Ni-MOF and Fe-MOF) were also synthesized, and their peroxidase-like activities for different substrates were investigated. Ni-MOF could not catalyze the oxidation of TMB, ABTS, OPD, and DAB, meaning that Ni-MOF had no peroxidase-mimicking activity (Fig. 6i and Supplementary Fig. 10). In contrast, Fe-MOF could rapidly catalyze the oxidation of the above four substrates, indicating Fe-MOF had excellent peroxidase-mimicking activity but no substrate selectivity (Fig. 6i and Supplementary Fig. 11). The above results reveal that the frame structure and the metal ions of MOF nanozymes were the prerequisites to its peroxidase-mimicking activity.

**Fig. 7:**
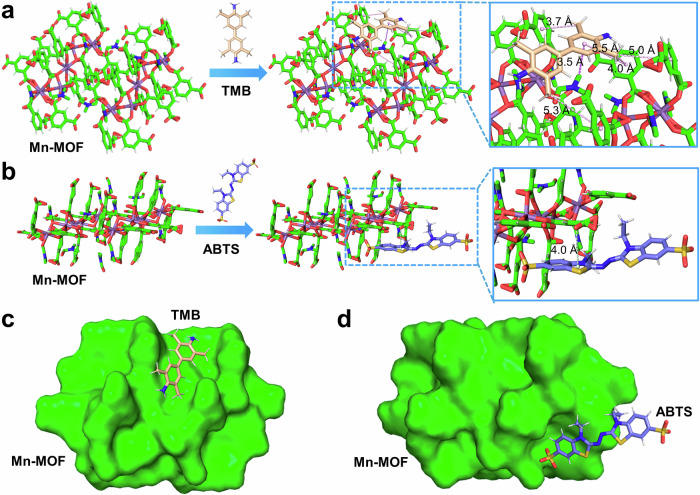
Molecular docking analysis of Mn-MOF with TMB and ABTS. Predicted binding modes of Mn-MOF with (**a**) TMB and (**b**) ABTS. Surface representation of the docked conformations for Mn-MOF complexed with (**c**) TMB and (**d**) ABTS. Purple and pink dotted lines denote Pi-sigma and multiple alkyl/Pi-alkyl hydrophobic interactions, respectively.

To further clarify the possible catalytic mechanism between Mn-MOF and TMB, we performed molecular docking simulations to investigate the interactions between Mn-MOF and two representative substrates: TMB and ABTS. As shown in Fig. 7a and Supplementary Table 6, the binding between Mn-MOF and TMB is predominantly mediated by hydrophobic interactions, including Pi-sigma and multiple alkyl/Pi-alkyl types. In contrast, only a weak Pi-anion interaction was observed between Mn-MOF and ABTS (Fig. 7b and Supplementary Table 6). Furthermore, the conformational analysis revealed that TMB is positioned within the internal structure of Mn-MOF (Fig. 7c), whereas ABTS remains at the peripheral region and cannot access the interior active sites (Fig. 7d). These results suggest that TMB exhibits stronger binding affinity and superior spatial compatibility with Mn-MOF, consistent with its higher catalytic activity.

### Keeping test strip from interferences of O_2_ and color by Mn-MOF nanozymes

**Fig. 8:**
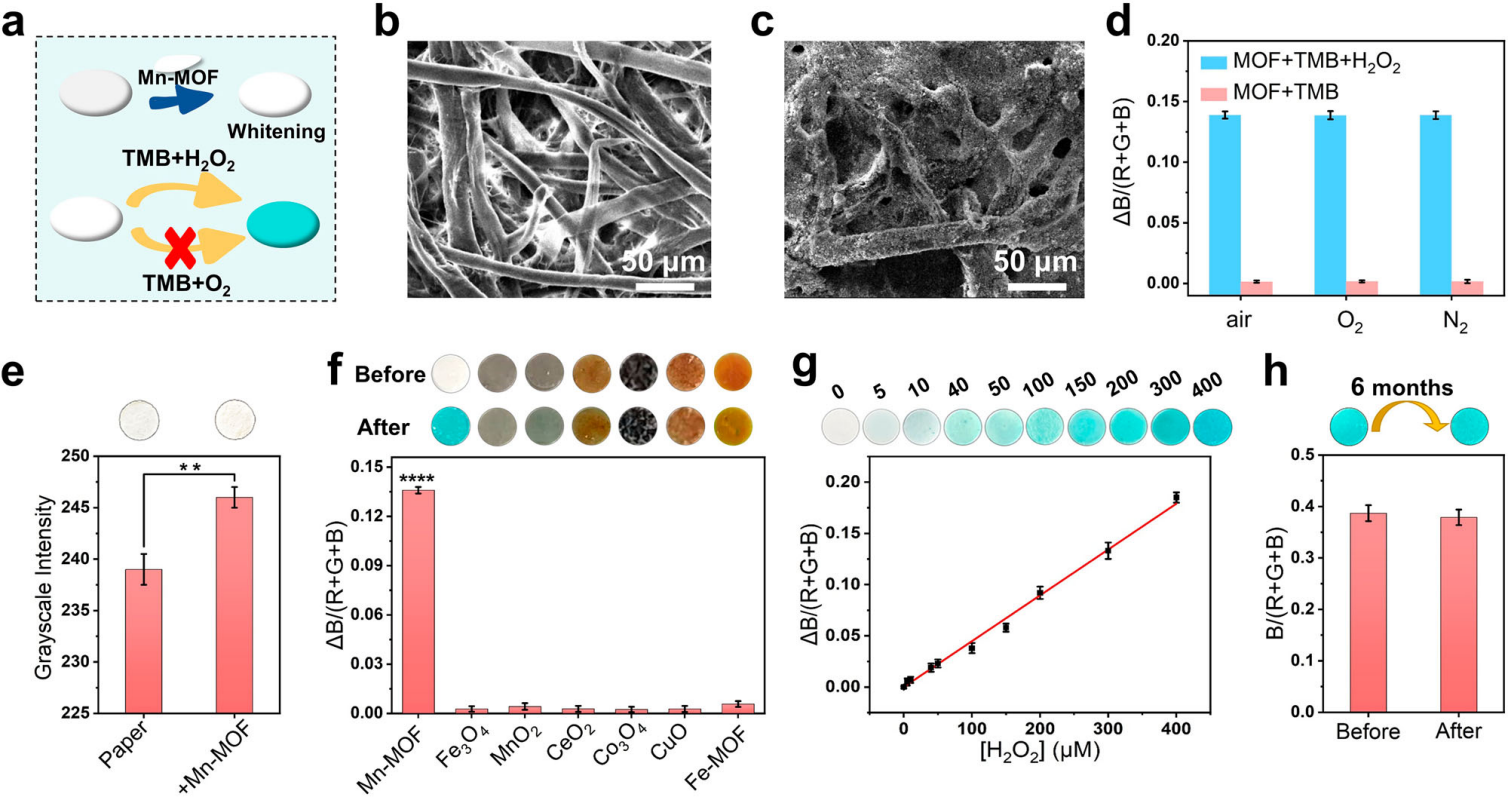
Colorimetric H_2_O_2_ detection by test strip based on Mn-MOF nanozymes. **a** Schematic of the proposed test strip. SEM images (**b**) before and (**c**) after the deposition of Mn-MOF nanozymes on paper. **d** Chromogenic response for H_2_O_2_ on paper based on Mn-MOF nanozymes in different atmospheres (air, O_2_, and N_2_). **e** Grayscale intensity of paper before and after the deposition of Mn-MOF nanozymes. **P < 0.001. **f** Photographs and ΔB/(R + G + B) values of test strip modified by Mn-MOF and other colored nanozymes. ****P < 0.0001. **g** Photographs of test strip for the detection of H_2_O_2_ and corresponding working curve of H_2_O_2_. **h** Chromogenic response of test strip after storage for 6 months. Error bars: mean ± SD (*n* = 3).

Nowadays, nanozymes-based test strip has shown great application potential for the rapid visual detection platform, due to the portability, low cost, high stability and easy large-scale production^18,20,21,79^. Here, considering the white color and the single activity of Mn-MOF nanozymes, we employed Mn-MOF nanozymes for the fabrication of test strip with TMB as chromogenic substrate, on the expectation that the two outstanding features would endow test strip some unique advantages (Fig. 8a). As a proof-of-concept, H_2_O_2_ test strip was constructed by deposition of Mn-MOF nanozymes on the paper (Fig. 8b, c). To prove the feasibility of the fabricated test strip, the H_2_O_2_ assay was performed in various atmospheres. As expected, the colorimetric result was not affected by the concentration of O_2_ (Fig. 8d), because of single peroxidase-like activity of Mn-MOF nanozymes without oxidase-like activity. Since the catalytic reaction cannot take place without H_2_O_2_, the reaction can be started only by adding H_2_O_2_, which prevents premature reactions in the air, and hence improves the reliability of the detection results.

More interestingly, commercial white papers are usually not very white, but Mn-MOF nanozymes showed whitening effect to the papers (Fig. 8e). This is crucial for enhancing sensitivity of the colorimetric result of test strip. To further confirm the superiority of white nanozymes to test strip, various colors of nanozymes in the same concentration were used to fabricate the nanozymes-based test strip. As shown in Fig. 8f, colored nanozymes masked the whiteness of paper and chromogenic reaction. In a sharp contrast, the blueness of chromogenic reaction on the paper modified by Mn-MOF nanozymes could be readily observed after the addition of H_2_O_2_. The above results demonstrate that white nanozymes with activity specificity were indeed an ideal choice for the colorimetric test strip based on nanozymes.

After the condition optimization (Supplementary Fig. 12), test strip was fabricated for the detection of H_2_O_2_, where colorless TMB could be oxidized into blue TMB*^+^ by H_2_O_2_ under the catalysis of Mn-MOF nanozymes. As shown in Fig. 8g, blueness of paper deepened with the increase of H_2_O_2_ concentration, indicating the constructed test strip can realize the fast qualitative assay of H_2_O_2_ by naked eye. Further, the corresponding RGB values were collected by smartphone for the quantitative analysis of H_2_O_2_. With the increase of concentration of H_2_O_2_, the value of ΔB/(R + G + B) continuously increased and showed a good linear relationship within the range of 5–400 μm. The regression equation was *y* = 0.00044731*x* + 0.000048 (*R*^2^ = 0.995). The limit of detection (LOD) was calculated to be 0.43 μm (S/*N* = 3), which was lower than some other methods including spectrophotometry and even electrochemical sensor (Supplementary Table 7)^80–83^, confirming the excellent sensitivity. In addition, test strip based on Mn-MOF nanozymes showed high storage stability, and there is not obvious change in the chromogenic response after the storage for 6 months at 4 °C (Fig. 8h).

So far, known nanozymes generally various colors, which seriously disturbs colorimetric analysis results of test strip, as proven by Fig. 8f. In this work, the white nanozymes did not disturb the colorimetric results of test strip, and highlighted the blue color of TMB*^+^, which resulted in high sensitivity of test strip. What’s more, Mn-MOF nanozymes had no oxidase-mimicking activity, and thus the proposed assay strategy could not be influenced by the varying concentration of O_2_ in atmosphere and solution (Figs. 4c, 8d), enhancing the reliability and avoiding false positive results for test strip. In addition, Mn-MOF nanozymes also possessed the superior stability to natural peroxidases. Taken together, Mn-MOF is an ideal enzyme substitute on test strip, and test strip based on Mn-MOF nanozymes would have broad application prospects.

### Universality of test strip based on Mn-MOF nanozymes

**Fig. 9:**
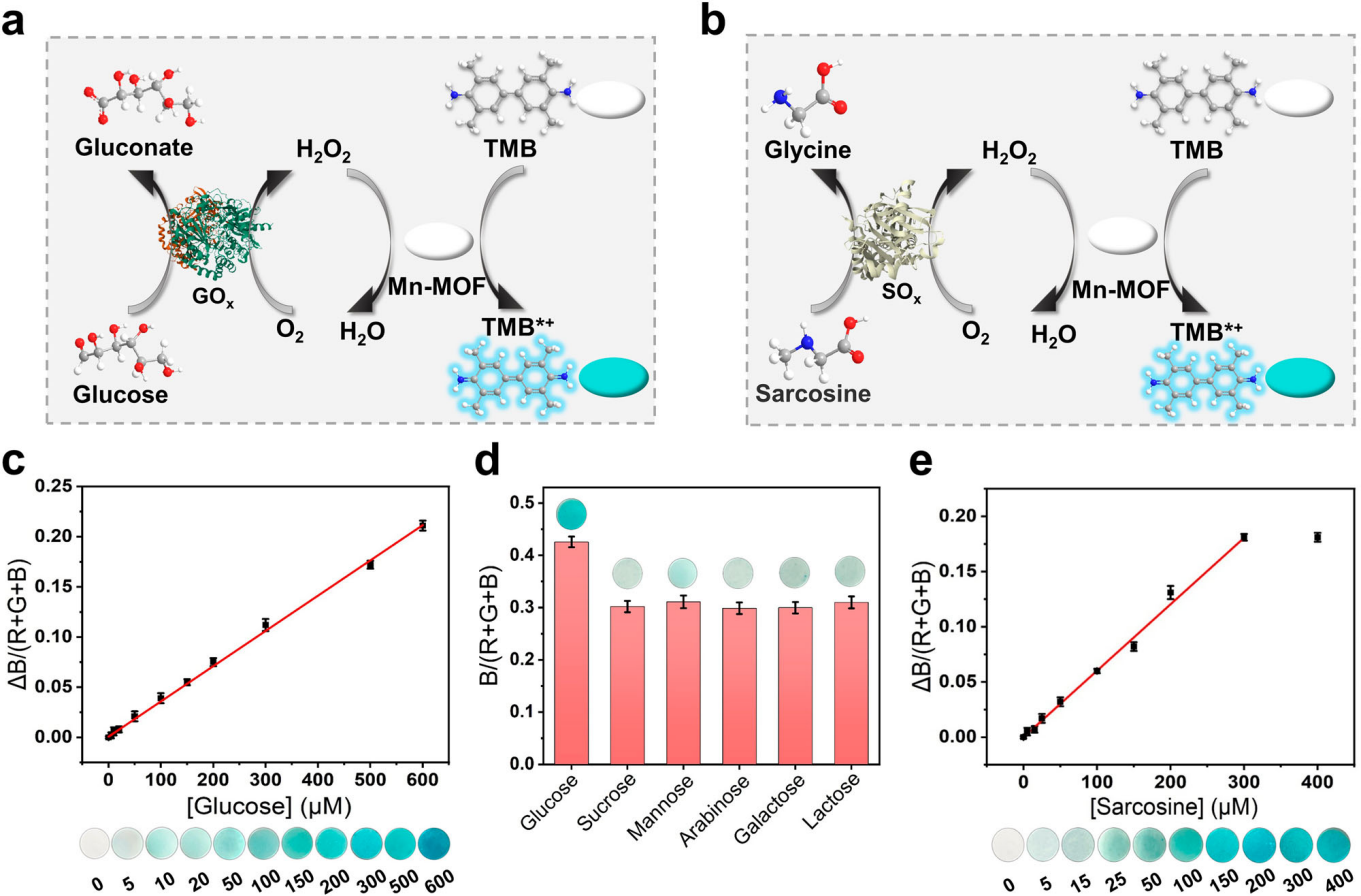
Universality of test strip based on Mn-MOF nanozymes. Schematic illustration of (**a**) glucose test strip, and (**b**) sarcosine test strip. **c** Photographs showing the response of glucose test strip to varying concentrations of glucose, and corresponding working curve for glucose detection. **d** Selectivity assay of glucose test strip. **e** Photographs of sarcosine test strip for different concentrations of sarcosine, and corresponding working curve for sarcosine detection. Error bars: mean ± SD (*n* = 3).

To prove the universality of Mn-MOF nanozymes-based test strip, glucose test strip was constructed by dropping glucose oxidase (GOx) onto Mn-MOF nanozymes-based test strip (Fig. 9a). In the GOx-nanozyme cascade catalytic system, glucose gave rise to the generation of H_2_O_2_, and then the chromogenic reaction. As the concentration of glucose gradually increased, the blue color on the paper gradually deepened, and the corresponding ΔB/(R + G + B) value gradually increased (Fig. 9c). Then, the calibration curve was plotted, and the regression equation was *y* = 0.00065347*x* + 0.000653 (*R*^2^ = 0.998) within the linear range of 5–600 μm. This method showed high sensitivity, and LOD (0.49 μm) was an order of magnitude lower than other colorimetric and electrochemical methods (Supplementary Table 8)^84–88^. In addition, we researched the selectivity of this system for other monosaccharides or disaccharides (sucrose, mannose, arabinose, galactose, and lactose). Only glucose showed a significant response, proving good selectivity of this system (Fig. 9d).

Subsequently, sarcosine test strip was also established by dropping sarcosine oxidase (SOx) onto the Mn-MOF nanozymes-modified paper (Fig. 9b). As the concentration of sarcosine gradually increased, the blue color on the paper gradually deepened, and the corresponding ΔB/(R + G + B) value gradually increased with a good linear correlation within 5–300 μm (Fig. 9e). The linear regression equation was fitted (*y* = 0.00060184*x* – 0.0000225, *R*^2^ = 0.998), and LOD was calculated to be 0.27 μm, which was lower than previous methods (Supplementary Table 9)^89–93^. These results suggested the versatility of Mn-MOF nanozymes-based test strip. Moreover, the sensitivity of test strip could be really improved by the whitening effect and the activity specificity of Mn-MOF nanozymes.

### Various assay applications in real samples

To estimate the applicability and reliability of the constructed test strip, the detection of H_2_O_2_, glucose and sarcosine in various real samples was conducted by three kinds of test strips. For H_2_O_2_ detection, two water-soak foodstuffs (sea cucumber and jellyfish) were used as real samples (Table 1). The concentrations of H_2_O_2_ were calculated according to the working curve (Fig. 7g). The standard addition method was applied, and the recoveries of H_2_O_2_were within 99.7–100.9%, suggesting good accuracy and reliability. The relative standard deviation (RSD) was within 3.75%, showing high reproducibility and precision.

**Table 1:** The determination of H_2_O_2_, glucose and sarcosine in real samples

Analyte	Sample	Spiked (μm)	Found^a^ (μm)	Recovery (%)	RSD^a^ (%, *n*= 5)
H_2_O_2_	Sea cucumber	0	5.60 ± 0.21	–	3.75
		10	15.57 ± 0.15	99.7	0.96
		150	156.43 ± 2.23	100.6	1.43
	jellyfish	0	12.80 ± 0.18	–	1.41
		10	22.89 ± 0.20	100.9	0.87
		150	162.74 ± 2.04	100.0	1.25
Glucose	apple	0	25.22 ± 0.19	–	0.75
		10	35.29 ± 0.25	100.7	0.71
		300	324.13 ± 2.43	99.6	0.74
	grape	0	30.15 ± 1.52	–	5.04
		10	40.04 ± 0.17	98.9	0.42
		300	332.27 ± 2.25	100.7	0.68
Sarcosine	beef	0	20.27 ± 0.24	–	1.18
		10	30.59 ± 0.12	103.2	0.39
		150	170.05 ± 3.03	99.9	1.78
	chicken	0	9.03 ± 0.19	–	2.10
		10	18.94 ± 0.27	99.1	1.43
		150	159.25 ± 2.34	100.1	1.47

^a^For each sample, experiment was repeated five times and ‘±’ represents SD (*n* = 5).

For the glucose detection in real samples, two fruits (apple and grape) were used as real samples (Table 1). After adding a known amount of glucose into the pretreated fruit juices, the recoveries of glucose were calculated to be from 98.9% to 100.7%. RSD was within 5.04% (*n* = 5). The results suggested the fabricated glucose test strip had a good accuracy and reproducibility.

For the detection in real samples, the reliability of proposed sarcosine test strip was also verified by measuring sarcosine in two meat samples (beef and chicken). The recoveries ranged from 99.1% to 103.2%, and RSD was within 2.10% (*n* = 5) (Table 1), indicating good reliability for the real samples assay.

## Discussion

In this work, white Mn-MOF nanozymes with good activity specificity were successfully synthesized by a simple ultrasound approach. Through comprehensive studies, we conclusively determined that the white optical property of Mn-MOFs originates exclusively from the specific coordination polymerization between Mn²⁺ and TPA ligands, independent of morphological characteristics, defect states, solvent effects, or specular reflection. The unique Mn-TPA combination exhibits negligible visible light absorption and generates high diffuse reflectance, resulting in its white appearance.

Noticeably, Mn-MOF demonstrated two types of catalytic specificity. (1) Reaction specificity: it possessed single peroxidase-mimicking activity without oxidase-like activity, thus avoiding O_2_ interference during the detection. (2) Substrate specificity: it specifically catalyzed the oxidation of TMB rather than other substrates (ABTS, OPD and DAB). These characteristics were innate and do not need any additional functional modification.

Significantly, white Mn-MOF nanozymes exhibited great superiority in colorimetric test strip for the apparatus-free visual detection of H_2_O_2_. Compared with other colors of nanozymes, the white Mn-MOF nanozymes avoided the interference with the judgment of the chromogenic results by the naked eye and the color extractor. Moreover, the whiteness of Mn-MOF strengthened the whiteness of paper, making the blue color of TMB*^+^ readily distinguishable, resulting in ultra-high sensitivity. On the basis of H_2_O_2_ test strip, a series of test strips was successfully developed for the detection of glucose and sarcosine, demonstrating excellent universality of Mn-MOF-based test strips. These test strips also showed high store stability for at least 6 months, and good reliability for real samples assay.

In summary, this work establishes a fundamental paradigm for understanding the origin of whiteness in MOF-based nanozymes through systematic optical and structural characterization. By elucidating the critical role of metal-ligand coordination in governing light absorption and scattering properties, we provide a design framework for developing white nanozymes with tailored optical behaviors. These findings directly address the long-overlooked challenge of color interference in nanozyme applications. As for the analysis technology, this work not only provides an ideal biomimetic recognition element (white nanozyme with single activity), but also presents an ideal vehicle (test strip) that can perfectly exploit the advantages of white nanozyme. We expect that this study would inspire the exploration of more and more white nanozymes with high activity specificity for various colorimetric assay methods (not limited to test strip), eliminating the interference of O_2_ and color of nanozymes. While nanozyme-based paper sensors integrate the advantages of nanozymes and paper substrates, challenges remain in transitioning from academic research to commercial applications. Key limitations include batch-to-batch reproducibility and the need for nanozymes with higher specificity to expand detection capabilities. Looking forward, the marriage of tunable MOF optics with enzymatic specificity opens transformative possibilities. These potential nanozymes could revolutionize low-cost, portable monitoring platforms.

## Methods

### Chemicals and materials

MnCl_2_·4H_2_O, OPD, TMB, ABTS, DAB and H_2_O_2_ were purchased from Sinopharm Chemical Reagent Co, Ltd. (China). BDC was purchased from Alfa Aesar (China). TEA, DMF and DCFH-DA were bought from Aladdin Reagent. GO_X_ and SO_X_ were purchased from Sigma Aldrich. All other reagents were of analytical grade and used without further purification. All aqueous solutions were prepared with ultrapure water from Milli-Q system (18.2 MΩ cm).

### Apparatus

The morphology of Mn-MOF nanozymes was characterized by TEM H-7650 (Hitachi, Japan) and SEM S-4800 (Hitachi, Japan). EDX spectroscopy and mapping was recorded on a spectrometer Ultim Max 40 (Oxford, UK). AFM assay was conducted by an AFM Multimode 8 (Bruker, Germany). XRD patterns were recorded on XRD D8 Advance (Bruker, Germany). XPS was performed by a spectrometer K-Alpha (Thermo Fisher Scientific, USA). The absorption spectra and the time-dependent absorbance were recorded on a microplate reader SpectraMax M2e (Molecular Devices, USA). The fluorescence was measured by fluorescence spectrophotometer F-7000 (Hitachi, Japan). ESR measurements were carried out by ESR Spectrometer A300 (Bruker, Germany).

### Synthesis of Mn-MOF nanozymes

Mn-MOF was synthesized by ultrasound approach. Firstly, TPA (0.75 mmol) was dissolved into the mixed solution containing DMF (32 mL), ethanol (2 mL), and water (2 mL). Then, MnCl_2_·4H_2_O (0.375 mmol) was added and dissolved into the above solution. Subsequently, TEA (0.8 mL) was quickly injected into the above solution, and then stirred for 5 min to obtain a white suspension. Then, the mixed solution was continuously sonicated at 40 kHz for 8 h, and the white product was obtained after centrifugation (4000 rpm), washing with ethanol, and drying in vacuum.

### Origin of whiteness in Mn-MOF

As for surface roughness, 3D topographic image of Mn-MOF was acquired, and then surface roughness parameters (*R*_a_ and *R*_q_) were measured by AFM assay (Bruker, USA). To eliminate the possibility of defect-state luminescence, the fluorescence spectrum of Mn-MOF was recorded by fluorescence spectrophotometer (Hitachi F-7000) with excitation at 253 nm (slit width: 10 nm) and emission scanned from 275 to 800 nm (slit width: 10 nm). To compare colorimetric parameters of different materials, their L*, a*, b*, *WI*, and Δ*E* values were conducted by a colorimeter LS171 (Linshang, China). The UV–Vis DRS of Mn-MOF powder was conducted on a spectrometer 3600-plus (Shimadzu, Japan) using diffuse reflection accessory adapted for powder samples. Then, these reflectance values were converted to Tauc plot, and the bandgap energy was assessed by Tauc plot, according to the Kubelka-Munk function^94^.

### Peroxidase-like activity assay of Mn-MOF nanozymes

Typically, the peroxidase-like activity of Mn-MOF was assessed by the characteristic absorbance of TMB*^+^(TMB oxide) at 652 nm (*ε*_652 nm_ = 39000 m^−1^ cm^−1^). H_2_O_2_ (1 mm) and TMB (0.5 mm), and Mn-MOF (50 μg mL^-1^) were reacted in the acetate buffer (0.1 m, pH 4.0) at 25 °C for 30 min. Subsequently, the absorption spectra and the time-dependent absorbance at 652 nm were recorded.

The effect of pH and temperature on peroxidase-like activity of Mn-MOF nanozymes was researched by the typical activity assay method, except for changing pH (pH 2.0–10.0) or temperature (4–50 °C). The thermal stability of Mn-MOF was analyzed by repeating the above experimental steps after incubating Mn-MOF at different temperatures (4–50 °C) for an hour. The thermal stability of Mn-MOF nanozymes were measured by the typical activity assay after Mn-MOF nanozymes were incubated at different various temperatures (4–90 °C) for an hour. Each experiment was repeated at least three times.

### Oxidase-like activity assay of Mn-MOF nanozymes

The analysis system of oxidase mimicking activity of Mn-MOF nanozymes was similar with the system of peroxidase-like activity, except that H_2_O_2_ was not added and the reaction was conducted under different atmospheres (air, O_2_, and N_2_) for 30 min.

### Steady-state kinetics assay

The steady-state kinetic analysis on the peroxidase-like activity of Mn-MOF nanozymes was performed by changing the concentration (0.05–1.2 mm) of TMB under fixed H_2_O_2_ concentration (1 mm) or varying the concentration (0.05–2 mm) of H_2_O_2_ at the constant TMB concentration (0.5 mm). To obtain Michaelis constant (*K*_m_), the activity data was fitted by the Michaelis-Menten equation: *ν* = *V*_max_ × [*S*]/(*K*_m_ + [*S*]), where *ν* is the initial velocity, *V*_max_ represents the maximum reaction velocity, and [*S*] represents the substrate concentration.

In order to explore the kinetic mechanism, the peroxidase-like activities were measured in the presence of the different concentrations of H_2_O_2_ (0.3, 0.6, 1 mm) or TMB (0.3, 0.5, 0.7 mm). The activity data were plotted based on the Lineweaver-Burk double reciprocal curve.

### Chemical ROS assay

In order to explore whether ROS were generated during the catalytic process, TA and DCFH-DA were used as fluorescent probes. Hydroxyl radicals (·OH) were detected by TA, where the reaction product, 2-hydroxy terephthalic acid, has an emission peak at 425 nm. H_2_O_2_ (1 mm) and TA (0.5 mm) were reacted with or without Mn-MOF (50 μg mL^−1^) in acetate buffer (0.1 m, pH 4.0) for 30 min at 25 °C. Subsequently, the fluorescence emission of the reaction solution was detected at 425 nm.

Total ROS were detected by DCFH, where the reaction product has an emission peak at 524 nm. Firstly, DCFH-DA (1 mm in MeOH, 1 mL) was hydrolyzed in NaOH aqueous solution (0.01 N, 4 mL) for 30 minutes to obtain the unstable DCFH. Then, the above solution was neutralized with phosphate solution (25 mm, 20 mL). Mn-MOF (10 mg mL^-1^, 20 μL), and 180 μL of fresh DCFH solution (40 μm) were mixed, and the fluorescence intensity at 524 nm was measured over time.

ESR measurements were carried out on ESR Spectrometer A300 (Bruker, Germany) to detect ROS (·OH, O₂·^−^, and ¹O₂) under ambient conditions. For ·OH and O₂·^−^ detection, DMPO was introduced into the buffer solution (pH 4.0) containing H₂O₂ and Mn-MOF. For ¹O₂ detection, TEMP was used under identical reaction conditions. The reaction system was evenly mixed and then transferred to a quartz tube, and measurements were taken at 0 min and 10 min in the dark.

### Molecular docking of Mn-MOF with substrate

The molecular structures of TMB, ABTS and the Mn-MOF model were initially prepared and optimized using MGL Tools 1.5.7, with polar hydrogens added and torsion degrees of freedom set appropriately. Docking simulation calculations were carried out using AutoDock 4.2. Docking procedures were carried out using the Triangle Matcher algorithm with the London dG scoring function for initial ligand placement, retaining 30 conformations for refinement. Subsequently, the conformational refinement was performed using the Rigid Receptor method with the GBVI/WSA dG scoring function. Visualization and structural interpretation of the binding interactions were conducted using PyMOL 2.5^79^.

### Construction of different test strips based on Mn-MOF nanozymes

For the typical construction of test strip for H_2_O_2_ assay, 5 μL of Mn-MOF (10 mg mL^-1^) and 10 μL of the acetate buffer solution (0.1 m, pH 4.0) containing TMB (0.5 mm) were successively dropped onto the filter paper (6 mm diameter), and air-dried to obtain the H_2_O_2_ test strip.

For the construction of test strip for glucose and sarcosine, the procedure is the same as that of H_2_O_2_ test strip, except for adding enzymes (GOx or SOx, 20 μg mL^−1^) into the acetate buffer solution (0.1 m, pH 4.0) containing TMB (0.5 mm).

### Detection of H_2_O_2_, glucose, and sarcosine by different test strips

For H_2_O_2_ detection, the different concentrations of H_2_O_2_ solutions (5 μL) were gently dropped onto the surface of test strip. After standing at 25 °C for 10 min, the RGB values of test strip were collected by Color Picker APP in smartphone for further quantitative analysis. For the detection of glucose and sarcosine, the different concentrations of glucose and sarcosine solutions (5 μL) were dropped onto the surface of the corresponding test strip. After standing at 25 °C for 30 min, the RGB values of test strip were collected for further quantitative analysis. The experiments were repeated three times, and LOD was calculated by the equation LOD = (3*σ*/*s*), where *σ* is the SD of blank signals and *s* is the slope of the calibration curve.

### Detection in various real samples

To demonstrate the applicability of the constructed test strip, various actual samples (including sea cucumber and jellyfish for H_2_O_2_ detection, apple and grape for glucose detection, and chicken and beef for sarcosine detection) were employed. For the pretreatment of actual samples, soak solutions of water-soaked foodstuffs (cucumber and jellyfish) were centrifuged for 15 min. Real glucose solutions were extracted by the homogenate of fruits (apple and grape), and the filtration with a 0.22 filter. The real sarcosine solutions were extracted by ultrasonication of meats (chicken and beef, 5 g) in acetate buffer (pH 4.0, 10 mL) for 30 min. The above obtained solutions were detected by the corresponding test strips.

### Statistical analysis

Each experiment was conducted with at least three independent replicates. Mean ± SD was calculated for each test. Unless otherwise specified, graphical data represent mean values from triplicate measurements. Statistical analysis was performed using GraphPad Prism Version 9.5.1 (GraphPad Software, USA). Significance was determined by one-way analysis of variance (ANOVA) followed by Tukey’s multiple comparison tests, with a two-tailed *P* values ≤ 0.05 considered significant. Statistical significance was denoted as follows: *P* ≤ 0.05 (*), *P* ≤ 0.01 (**), *P* ≤ 0.001 (***), *P* ≤ 0.0001 (****). *P* > 0.05, not significant (ns).

## Supplementary information

Supplementary Information

## Data Availability

All the data that support the findings of this study are available within the article and Supplementary Information file, or from the corresponding authors upon request.
