# Supplementary material for: White Mn-MOF nanozymes with peroxidase-activity specificity overcome color and O2 effects on colorimetric test strips
Source: Commun Chem. 2025 Dec 4;8:389. doi: 10.1038/s42004-025-01772-z (PMC12678827; doi:10.1038/s42004-025-01772-z)
Supplement: Supplementary file 1 — Supplementary Information [file 42004_2025_1772_MOESM1_ESM.pdf]

Supplementary Information

White Mn-MOF nanozymes with peroxidase-activity specificity overcome color and O_2 effects on colorimetric test strips

Lei Han[1]*, Jingying Tan[1], and Yucui Zhang[1]

[1]*College of Chemistry and Pharmaceutical Sciences, Qingdao Agricultural University, 700 Changcheng Road, Qingdao 266109, Shandong, China.*

[*] Corresponding author.

E-mail address: hanlei@qau.edu.cn; ORCID: 0000-0003-1955-0718

Contents

Supplementary Table 1. Statistical analysis of length and width dimensions for Mn-MOF based on TEM measurements……………………………………………………………S3
Supplementary Fig. 1. Size distribution histogram of Mn-MOF NSs……………………S3
Supplementary Fig. 2. TEM image of bulk Mn-MOF………………………………………S4
Supplementary Fig. 3 XPS spectra of Mn-MOF nanozymes………………………………S5
Supplementary Table 2. Elemental composition analysis of Mn-MOF based on XPS……..S6
Supplementary Table 3. Colorimetric parameters of different materials……………………S7
Supplementary Fig. 4. Characterization for POD-like activity of Mn^{2+} and TPA……………S8
Supplementary Fig. 5. The column graphs of absorbance of TMB oxidation by different concentrations of Mn-MOF nanozymes with H_2O_2……………………………………………S8
Supplementary Fig. 6. Peroxidase-like activity and photographs of Mn-MOF nanozymes before and after 6 months of storage………………………………………………………S9
Supplementary Fig. 7. Steady-state kinetics of Mn-MOF nanozymes……………………S10
Supplementary Table 4. Comparison of K_m and V_{max} between Mn-MOF nanozymes and other catalysts…………………………………………………………………………………S11
Supplementary Fig. 8. Peroxidase-like activity of Mn-MOF nanozymes for different substrates……………………………………………………………………………………S12
Supplementary Fig. 9. Oxidase-like activity of Mn-MOF nanozymes for different substrates……………………………………………………………………………………S13
Supplementary Table 5. Comparison of specific activity among POD-like Mn-based nanozymes…………………………………………………………………………………S14
Supplementary Fig. 10. Peroxidase-like activity of Ni-MOF nanozymes…………………S15
Supplementary Fig. 11. Peroxidase-like activity of Fe-MOF nanozymes…………………S16
Supplementary Table 6. Bonding Distances corresponding to the distinct interactions between Mn-MOF and TMB or ABTS…………………………………………………S17
Supplementary Table 7. Comparison of various H_2O_2 detection methods…………………S18
Supplementary Fig. 12. The concentration optimization of Mn-MOF nanozymes on test strip…………………………………………………………………………………………S19
Supplementary Table 8. Comparison of various glucose detection methods………………S20
Supplementary Table 9. Comparison of various sarcosine detection methods……………S21
Supplementary References……………………………………………………………S22

Supplementary Table 1. Statistical analysis of length and width dimensions for Mn-MOF based on TEM measurements

**Size**	**Range (μm)**
Length	1.99–0.28
Width	1.46–0.18

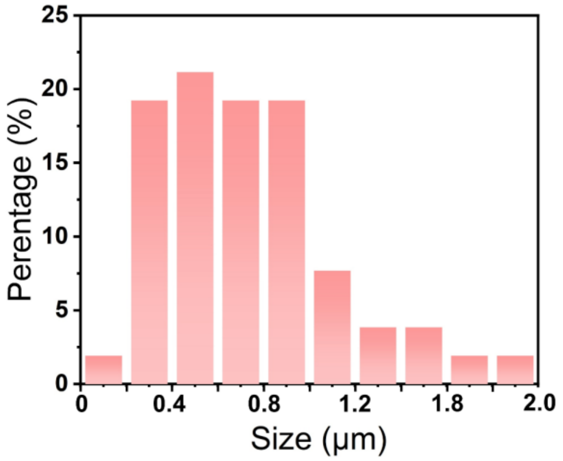

Supplementary Fig. 1. Size distribution histogram of Mn-MOF NSs. Size includes length and width.

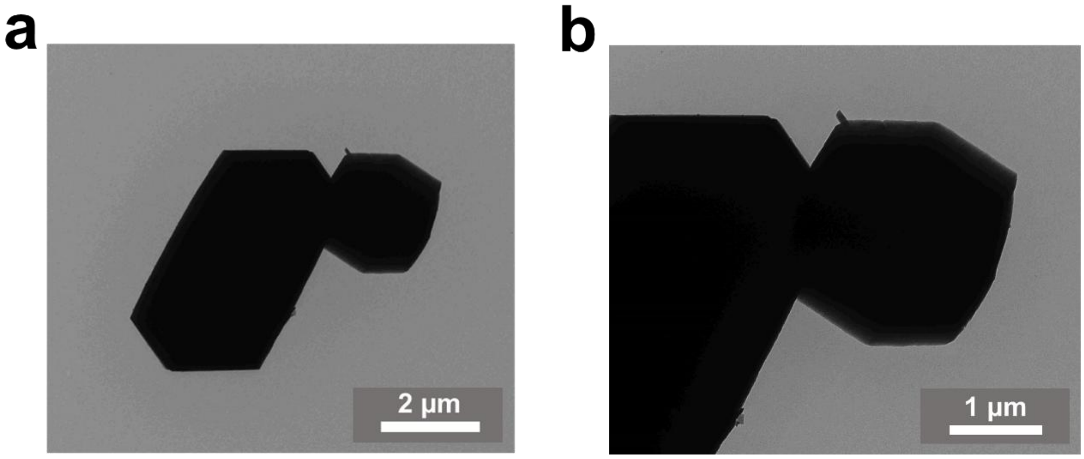

Supplementary Fig. 2. TEM image of bulk Mn-MOF.

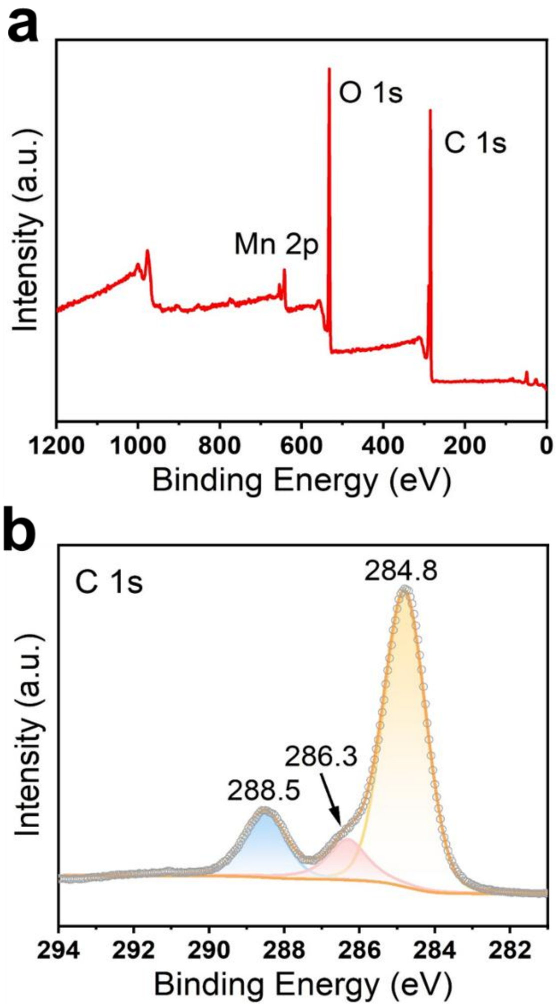

Supplementary Fig. 3. XPS spectra of Mn-MOF nanozymes. **(a)** The XPS full spectra, and **(b)** high-resolution patterns of C 1s of Mn-MOF nanozymes.

Supplementary Table 2. Elemental composition analysis of Mn-MOF based on XPS

Element	Peak BE (eV)[a]	Height (CPS)[b]	FWHM (eV)[c]	Atomic (%)
Mn 2p	642.2	32225.45	3.2	5.36
O 1s	531.6	120861.11	1.75	26.72
C 1s	284.8	125405.92	1.34	67.92

a) BE, binding energy;

b) CPS, counts per second;

c) FWHM, full width at half maximum.

Supplementary Table 3. Colorimetric parameters of different materials.

Sample [a]	CIE-$L^*a^*b^*$ parameters			WI [b]	ΔE [c]
	L^*	a^*	b^*		
Mn-TPA MOF	93.09	3.53	-0.53	93.78	0.65
Mn-ATPA MOF	69.51	10.15	7.59	46.74	23.15
Mn-TMA MOF	81.02	3.64	4.97	66.11	12.55
Co-MOF	67.38	11.52	3.45	57.03	25.36
Fe-MOF	36.45	3.39	-0.28	37.29	57.19
TiO_2	93.80	-0.70	0.20	93.20	–

a) Colorimetric parameters of TiO_2 were obtained from reference [1].

b) $WI = L^* - 3b^*$

c) $\Delta E = \sqrt{(\Delta L^*)^2 + (\Delta a^*)^2 + (\Delta b^*)^2}$

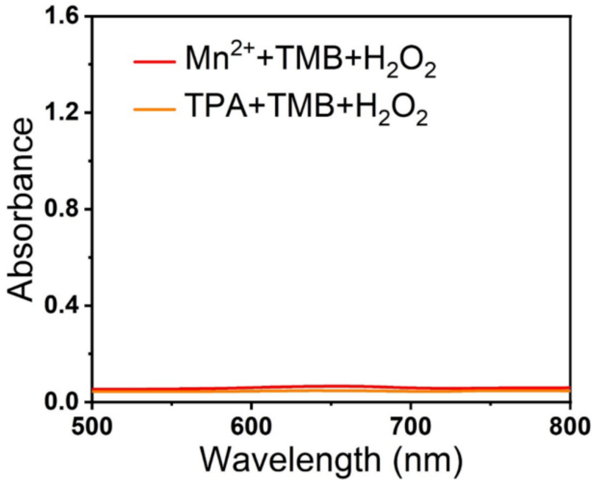

Supplementary Fig. 4. Characterization for POD-like activity of Mn^{2+} and TPA.

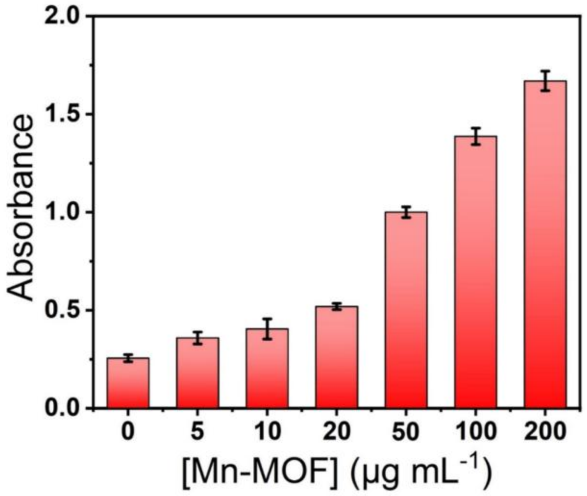

Supplementary Fig. 5. The column graphs of absorbance of TMB oxidation by different concentrations of Mn-MOF nanozymes with H_2O_2. The Absorbance of TMB with different concentrations of Mn-MOF nanozymes (0, 5, 10, 20, 50, 100 and 200 μg mL^{-1}) in the presence of H_2O_2 after incubation (0.1 M acetate buffer, pH 4.0). Error bars: mean ± SD (n = 3).

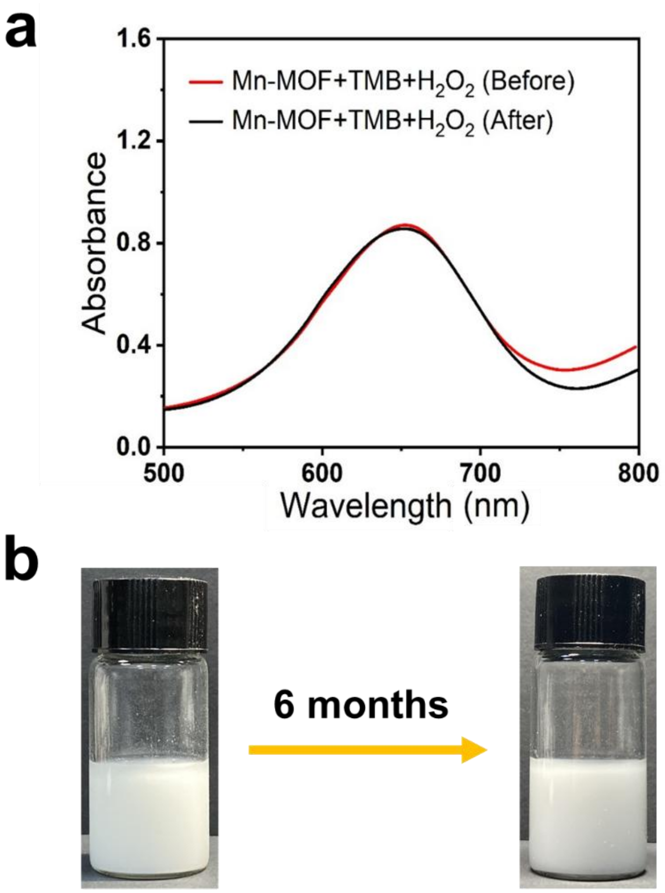

Supplementary Fig. 6. Peroxidase-like activity and photographs of Mn-MOF nanozymes before and after 6 months of storage.

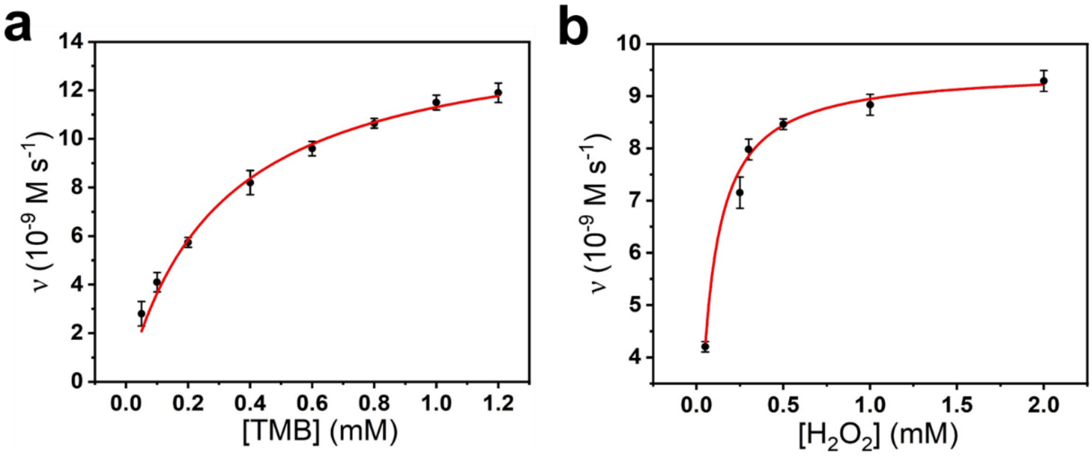

Supplementary Fig. 7. Steady-state kinetics of Mn-MOF nanozymes. Michaelis-Menten curves fit for **(a)** varied concentrations (0.05–1.2 mM) of TMB with fixed concentration (1 mM) of H_2O_2, and **(b)** varied concentrations (0.1–2 mM) of H_2O_2 with fixed concentration (0.5 mM) of TMB. Error bars: mean ± SD (n = 3).

Supplementary Table 4. Comparison of K_m and V_{max} between Mn-MOF nanozymes and other catalysts.

Catalyst[a]	Substrate	K_m (mM)	V_{max} (10^{-8} M s^{-1})	Ref.
HRP	TMB	0.434	10.0	[2]
	H_2O_2	3.700	8.71	
Fe_3O_4	TMB	0.098	3.44	[2]
	H_2O_2	154	9.78	
$NiFe_2O_4$	TMB	0.550	4.57	[3]
	H_2O_2	2.600	14.11	
Co_3O_4	TMB	0.037	6.27	[4]
	H_2O_2	140.07	12.1	
CuO	TMB	25	10.49	[5]
	H_2O_2	400	16.1	
Ni-MOF nanosheet	TMB	0.365	6.53	[6]
	H_2O_2	2.490	130	
Ni-MOF	TMB	0.431	3.10	[7]
	H_2O_2	0.277	3.25	
Au/Co_3O_4-CeO_x	TMB	0.122	0.87	[8]
	H_2O_2	0.272	0.40	
GeO_2	TMB	0.420	23.40	[9]
	H_2O_2	1.750	23.40	
Mn-MOF	TMB	0.310	1.48	This work
	H_2O_2	0.063	0.95	

a) HRP, horse-radish peroxidase.

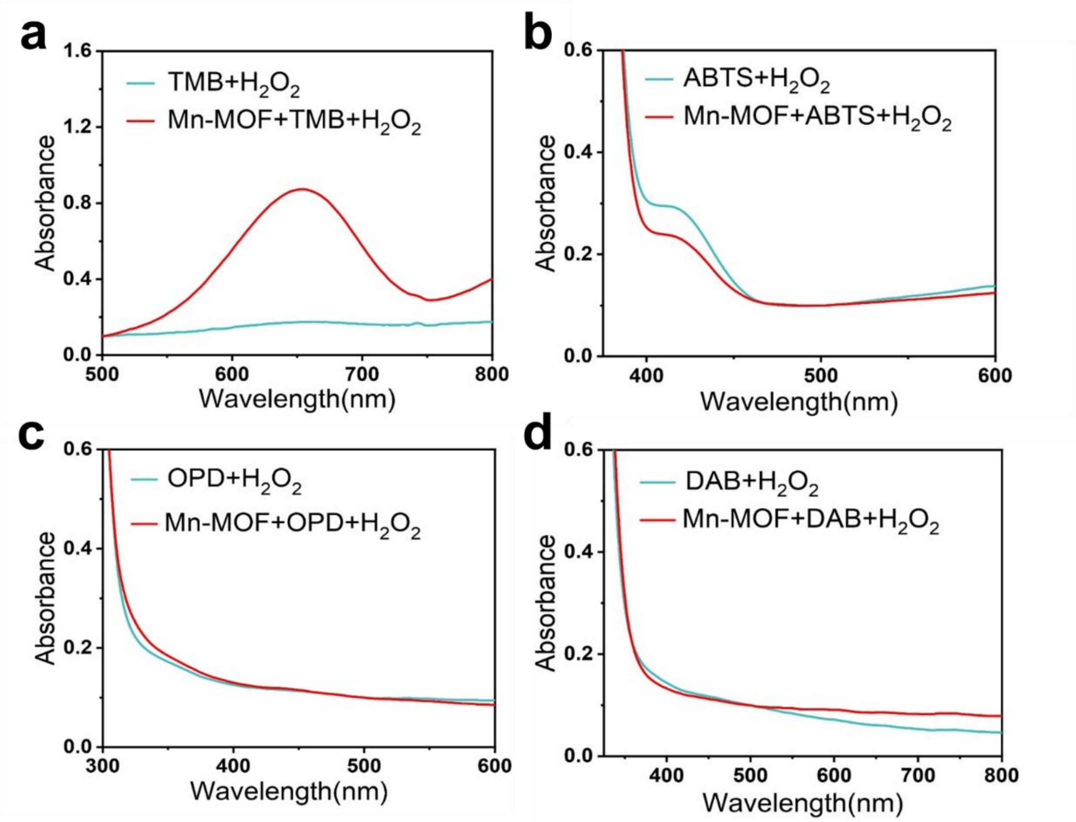

Supplementary Fig. 8. Peroxidase-like activity of Mn-MOF nanozymes for different substrates. Typical absorption spectra of **(a)** TMB, **(b)** ABTS, **(c)** OPD and **(d)** DAB oxidation catalyzed by Mn-MOF nanozymes and controls in the presence of H_2O_2 in the acetate buffer (pH 4.0, 0.1 M).

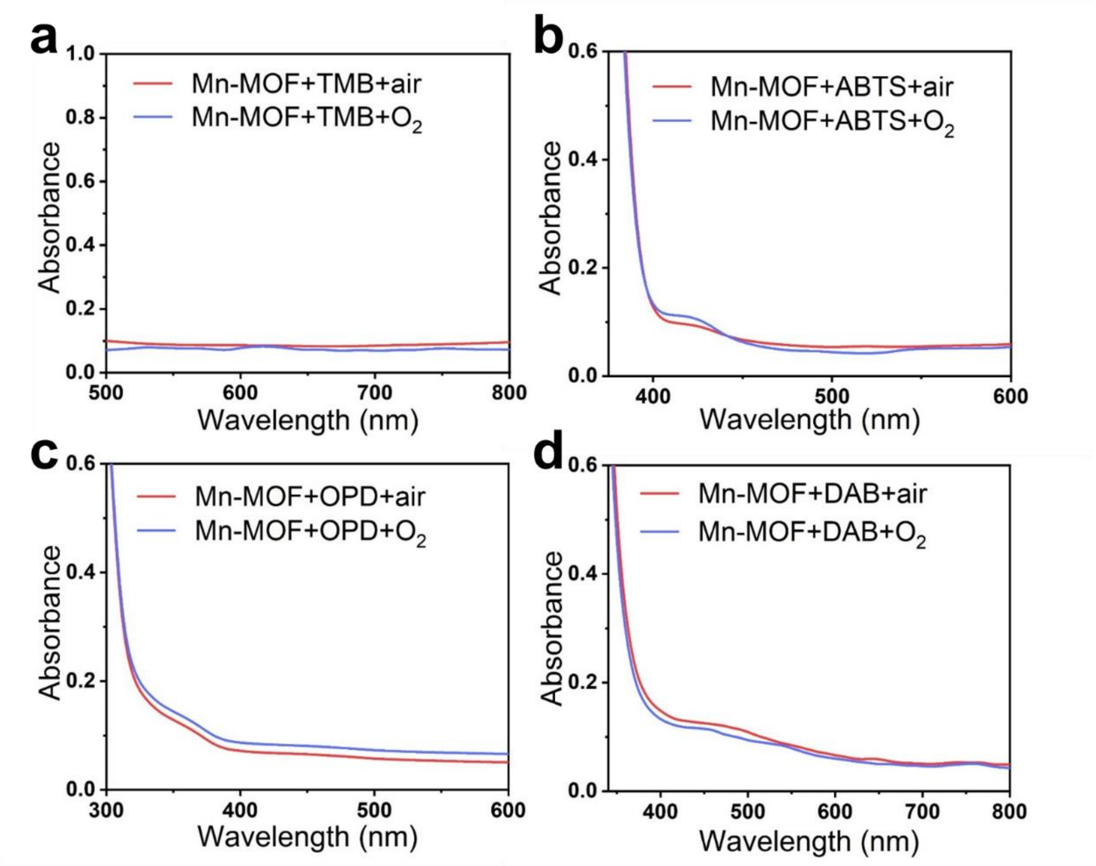

Supplementary Fig. 9. Oxidase-like activity of Mn-MOF nanozymes for different substrates. Absorption spectra of catalytic oxidation of **(a)** TMB, **(b)** ABTS, **(c)** OPD and **(d)** DAB by Mn-MOF nanozymes under air, O_2 atmosphere.

Supplementary Table 5 . Comparison of specific activity among POD-like Mn-based nanozymes

**Materials**[a]	**Specific activity (U mg^{-1})**	**Ref.**
Mn/PSAE	0.00504	[10]
MnO_2 NPs	0.00091	[10]
Mn-MOF	0.03175	This work

a) PSAE, polyethylene glycolylated single-atom enzyme.

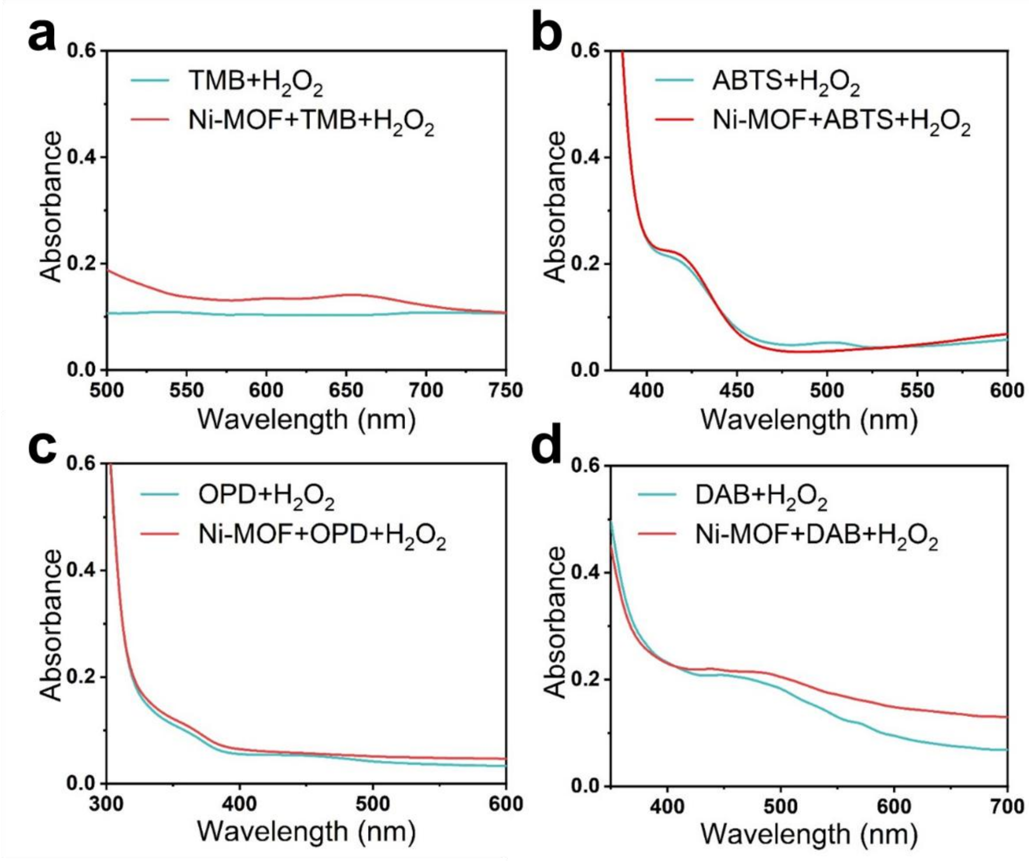

Supplementary Fig. 10. Peroxidase-like activity of Ni-MOF nanozymes. Absorption spectra of catalytic oxidation of **(a)** TMB, **(b)** ABTS, **(c)** OPD and **(d)** DAB by Ni-MOF nanozymes.

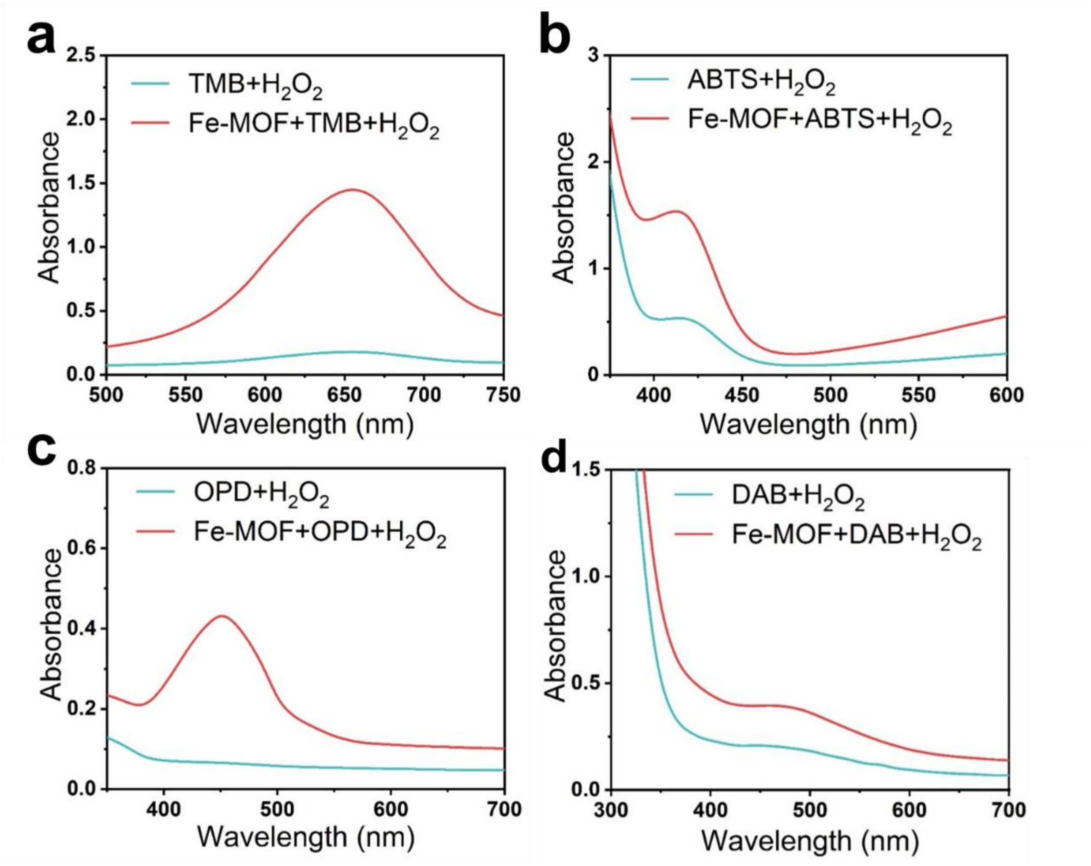

Supplementary Fig. 11. Peroxidase-like activity of Fe-MOF nanozymes. Absorption spectra of catalytic oxidation of **(a)** TMB, **(b)** ABTS, **(c)** OPD and **(d)** DAB by Fe-MOF nanozymes.

Supplementary Table 6. Bonding Distances corresponding to the distinct interactions between Mn-MOF and TMB or ABTS.

**Substrate**	**Type of interaction**	**Distance (Å)**
TMB	Pi-Sigma	3.5
	Alkyl	3.7
	Alkyl	4.0
	Alkyl	5.0
	Alkyl	5.3
	Pi-Alkyl	5.5
ABTS	Pi-Donor Hydrogen Bond	4.0

Supplementary Table 7. Comparison of various H_2O_2 detection methods

**Method**[a]	**Materials**[b]	**Linear rang**	**LOD**	**Ref.**
Electrochemistry	FeMo	1–100 μM	0.81 μM	[11]
Electrochemistry	FePr	10–110 μM	4.9 μM	[12]
Electrochemistry	PBEA	5–1000 μM	1.9 μM	[13]
HSV<br>Chromometry	MOF-818	0.033–66 mM<br>0.0133–10 mM	15.37μM<br>9.02 μM	[14]
Chromometry	Mn-MOF	5–400 μM	0.43 μM	This work

a) HSV, hue-saturation-value;

b) PBEA, prussian blue-based electrode array.

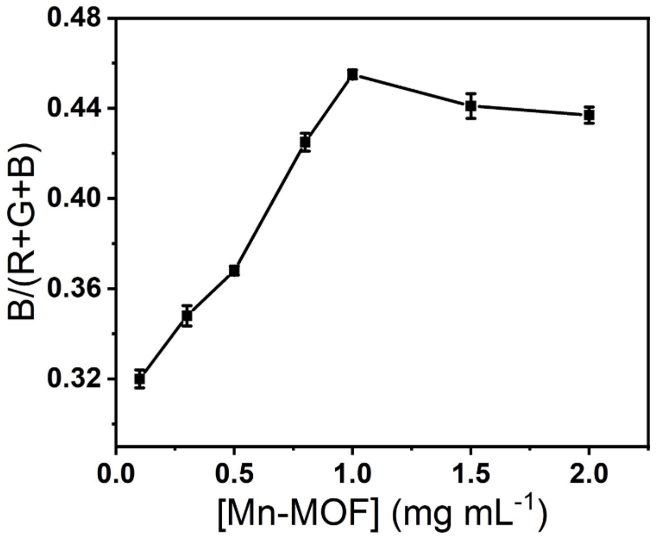

Supplementary Fig. 12. The concentration optimization of Mn-MOF nanozymes on test strip.

Error bars: mean ± SD (n = 3).

Supplementary Table 8. Comparison of various glucose detection methods.

Method	Materials[a]	Linear rang	LOD	Ref.
Electrochemistry	PGO_x@MXene/CS	0.03–16.5 mM	3.1 μM	[15]
Electrochemistry	CoOOH@Cu	0.025–2.5 mM	7.38 μM	[16]
Chromometry		0.025–2.5 mM	2.07 μM	
Chromometry	$TiSe_{2-x}$@Au	0.015–0.6 mM	3.7 μM	[17]
Chromometry	PGA-Fe/CS	20–600 μM	2.4 μM	[18]
Chromometry	Fe SSN	10–60 μM	2.1 μM	[19]
Chromometry	Mn-MOF	5–600 μM	0.49 μM	This work

[a)] PGOx, GOx polynanogel; CS, chitosan; PGA, poly-γ-glutamic acid; Fe SSN, single iron site nanozymes.

Supplementary Table 9. Comparison of various sarcosine detection methods.

Method	Materials[a]	Linear rang	LOD	Ref.
Electrochemistry	$CuInS_2$	10–1000 μM	8.0 μM	[20]
Fluorescence	CuT@N/Au	10–1100 μM	4.7 μM	[21]
Chromometry	Fe_3O_4@SiO_2@$NiCo_2S_4$	1.25–350 μM	0.42 μM	[22]
Chromometry	NQS/GO	6.2–263 μM	0.73 μM	[23]
Chromometry	Fe-doped g-C_3N_4	10–500 μM	3.6 μM	[24]
Chromometry	Mn-MOF	5–300 μM	0.27 μM	This work

[a)] CuT, tryptophan-based Cu-containing; NQS, 1,2-naphthoquinone-4-sulphonic acid sodium salt; GO, graphene oxide.

Supplementary References

[1] Wang, Y., Mumford, K. A., Shen, S. & Li, Y. *Powder Technology* **2015**, 284, 204-209.

[2] L. Gao, J. Zhuang, L. Nie, J. Zhang, Y. Zhang, N. Gu, T. Wang, J. Feng, D. Yang, S. Perrett, X. Yan, *Nature Nanotechnology* **2007**, 2, 577.

[3] L. Su, W. Qin, H. Zhang, Z. U. Rahman, C. Ren, S. Ma, X. Chen, *Biosensors and Bioelectronics* **2015**, 63, 384.

[4] J. Mu, Y. Wang, M. Zhao, L. Zhang, *Chemical Communications* **2012**, 48, 2540.

[5] J A. P. Nagvenkar, A. Gedanken, *ACS Applied Materials & Interfaces* **2016**, 8, 22301.

[6] Chen, Y. Shu, H. Li, Q. Xu, X. Hu, *Talanta* **2018**, 189, 254.

[7] J. Guo, Y. Liu, Z. Mu, S. Wu, J. Wang, Y. Yang, M. Zhao, Y. Wang, *Microchimica Acta* **2022**, 189, 219.

[8] H. Liu, Y. Ding, B. Yang, Z. Liu, Q. Liu, X. Zhang, *Sensors and Actuators B: Chemical* **2018**, 271, 336.

[9] X. Liang, L. Han, *Advanced Functional Materials* **2020**, 30, 2001933.

[10] Y. Zhu, W. Wang, J. Cheng, Y. Qu, Y. Dai, M. Liu, J. Yu, C. Wang, H. Wang, S. Wang, C. Zhao, Y. Wu, Y. Liu, *Angewandte Chemie International Edition* 2021, 60, 9480.

[11] H. Li, Q. Cai, J. Wang, G. Jie, *Biosensors and Bioelectronics* **2023**, 232, 115315.

[12] K. Niu, J. Chen, X. Lu, *Chemical Engineering Journal* **2023**, 475, 146491.

[13] D. Rojas, J. F. Hernández-Rodríguez, F. Della Pelle, M. Del Carlo, D. Compagnone, A. Escarpa, *Biosensors and Bioelectronics* **2020**, 170, 112669.

[14] K. Yu, M. Li, H. Chai, Q. Liu, X. Hai, M. Tian, L. Qu, T. Xu, G. Zhang, X. Zhang, *Chemical Engineering Journal* **2023**, 451, 138321.

[15] X. Tong, L. Jiang, Q. Ao, X. Lv, Y. Song, J. Tang, *Biosensors and Bioelectronics* **2024**, 248, 115942.

[16] H. Cheng, Z. Wang, H. Sun, B. Chen, J. Huang, R. Jia, X. He, K. Wang, *Chemical Communications* **2022**, 58, 13487.

[17] C. Sun, X. Zhang, H. Huang, Y. Liu, X. Mo, Y. Feng, J. Wang, W. Zhou, P. K. Chu, X.-F. Yu, W. Liu, *Biosensors and Bioelectronics* **2023**, 241, 115665.

[18] Q. H. Nguyen, D. H. Lee, P. T. Nguyen, P. G. Le, M. I. Kim, *Chemical Engineering Journal* **2023**, 454, 140541.

[19] M. Chen, H. Zhou, X. Liu, T. Yuan, W. Wang, C. Zhao, Y. Zhao, F. Zhou, X. Wang, Z. Xue, T. Yao, C. Xiong, Y. Wu, *Small* **2020**, 16, 2002343.

[20] X.-Y. Jiang, L. Zhang, Y.-L. Liu, X.-D. Yu, Y.-Y. Liang, P. Qu, W.-W. Zhao, J.-J. Xu, H.-Y. Chen, *Biosensors and Bioelectronics* **2018**, 107, 230.

[21] M. Wang, L. Zhang, X. Zhou, J. Zhang, C. Zhou, X. Su, *Analytica Chimica Acta* **2022**, 1223, 340188.

[22] X. Wang, M. Chen, L. Zhao, *Chemical Engineering Journal* **2023**, 468, 143612.

[23] Z. Xue, B. Yin, H. Wang, M. Li, H. Rao, X. Liu, X. Zhou, X. Lu, *Nanoscale* **2016**, 8, 5488.

[24] X. Xi, X. Peng, C. Xiong, D. Shi, J. Zhu, W. Wen, X. Zhang, S. Wang, *Microchimica Acta* **2020**, 187, 383.
